# Characterization of diet-linked amino acid pool influence on *Fusobacterium* spp. growth and metabolism

**DOI:** 10.1128/msphere.00789-24

**Published:** 2025-02-13

**Authors:** Avery V. Robinson, Sarah J. Vancuren, Massimo Marcone, Emma Allen-Vercoe

**Affiliations:** 1 Department of Molecular and Cellular Biology, University of Guelph, Guelph, Canada; 2 Department of Food Science, University of Guelph, Guelph, Canada; University of Michigan-Ann Arbor, Ann Arbor, Michigan, USA

**Keywords:** *Fusobacterium*, colorectal cancer, amino acid preference

## Abstract

The genus *Fusobacterium* contains multiple proteolytic opportunistic pathogens that have been increasingly linked to colorectal cancer (CRC). “Oncomicrobes” such as these fusobacterial species within the gut microbiota may contribute to CRC onset and/or progression. Protein-rich diets may both directly increase CRC risk and enrich for proteolytic oncomicrobes, including *Fusobacterium* spp. Individual food substrates vary in amino acid content, and released amino acid content that is not absorbed in the small intestine may influence the growth of colonic proteolytic fermenters. Fusobacteria such as *Fusobacterium* spp. are known to preferentially metabolize certain amino acids. As such, some foods may better support the growth of these species within the colonic environment than others. To explore this, in this study, we created free amino acid pools (FAAPs) to represent proportions of amino acids in major proteins of three common dietary protein sources (soy, beef, and bovine milk). Growth curves were generated for 39 *Fusobacterium* spp. strains cultured in a dilute medium supplemented with each of the three FAAPs. Thereafter, amino acid use by 31 of the 39 *Fusobacterium* spp. strains in each FAAP treatment was assessed. FAAP supplementation increased growth metrics of all *Fusobacterium* spp. strains tested; however, the strains varied greatly in terms of the FAAP(s) generating the greatest increase in growth. Furthermore, the amino acid utilization strategy was highly variable between strains of *Fusobacterium* spp. Neither growth metrics nor amino acid utilization could be explained by species classification of *Fusobacterium* spp. strains. This report expands upon the previous knowledge of fusobacterial amino acid metabolism and indicates that proteolytic oncomicrobial activity should be assessed in the context of available protein sources.

**IMPORTANCE:**

*Fusobacterium* spp. including *F. animalis*, *F. nucleatum*, *F. vincentii*, and *F. polymorphum* are common oral commensals with emerging importance in diseases across multiple body sites, including CRC. CRC lesions associated with fusobacteria tend to result in poorer prognosis and increased disease recurrence. While *Fusobacterium* spp. are thought to colonize after tumorigenesis, little is known about the factors that facilitate this colonization. Protein-rich diets yielding readily metabolized free amino acids within the colon may promote the growth of proteolytic fermenters such as fusobacteria. Here, we show that variable concentrations of free amino acids within pools that represent different dietary protein sources differentially influence fusobacterial growth, including CRC-relevant strains of *Fusobacterium* spp. This work highlights the high degree of variation in fusobacterial amino acid utilization patterns and suggests differing proportions of dietary amino acids that reach the colon could influence fusobacterial growth.

## INTRODUCTION

Diet, particularly protein and fiber substrates, has been linked to risk of development of human colorectal cancer (CRC). Fiber-rich diets can promote short-chain fatty acid production, particularly butyrate, within the colon, which can suppress colonic inflammation and tumorigenesis (1, 2). Butyrate may also inhibit histone deacetylase in cancerous colonocytes, resulting in dampening of inflammation and promotion of cancerous colonocyte apoptosis (3). While fiber has been reported to be protective against CRC, protein-rich diets—and, in particular, diets rich in red meat (4, 5)—can increase the risk of disease development (6). It has been proposed that *N*-nitroso compounds resulting from heme iron and/or nitrate metabolism (7) could cause a mutagenic signature in CRC (8).

Protein-rich diets may influence disease risk via enrichment of CRC-linked bacterial taxa and metabolites within gut bacterial communities (9). For example, a protein-rich diet may enrich for proteolytic oncomicrobes such as *Fusobacterium* spp. (10). One such fusobacterial species, *F. nucleatum*, was first linked to CRC when fusobacterial genomic and transcriptomic signatures were found to be enriched along colorectal lesions compared to adjacent healthy tissue (11, 12). *F. nucleatum* has since been shown to be capable of promoting intestinal tumorigenesis (13–15), chemoresistance, and disease recurrence (16, 17); preventing cancerous colonocyte apoptosis (18); lowering disease survival rates (19); and being retained within colorectal tumors during metastasis (20). *F. nucleatum* is a heterogenous species, and the subspecies “animalis,” “polymorphum,” and “vincentii” have been recently recognized as species in their own right (21).

It is recommended that a healthy human diet should contain between 0.8 g and to 1.6 g of protein per kg body weight per day (22), regardless of the source. Protein consumption cannot be avoided; this would result in nutrient deficiency and unwanted symptoms such as fatigue and irritability (23). We asked instead whether it might be possible to modify dietary protein sources to prevent or reduce fusobacterial proliferation within the human colon.

Free amino acids can reach the colon in gnotobiotic and conventional mice (24), suggesting not all amino acids that are released during food digestion are absorbed within the small intestine. *Fusobacterium* spp. utilize certain amino acids preferentially over others; for example, glutamate and histidine are commonly preferred fusobacterial metabolic substrates (25). Diets that are rich in *Fusobacterium*-preferred amino acids could, in theory, enrich for strains of this genus within the colon. While balanced omnivorous, vegetarian, and vegan diets tend to be largely similar in amino acid percent composition compared to total amino acids (26), individual foods vary widely in their relative proportions of amino acids (27–30).

Here, we applied a reductionist approach to determine whether amino acid proportions within certain foods might enrich for *Fusobacterium* spp. within the human colon. Three common dietary protein sources were selected: beef (specifically, bovine psoas major [filet mignon]), bovine milk, and soybean. Instead of analyzing hydrolysates of these protein types (which would be complicated by the presence of residual fats and/or carbohydrates), the amino acid compositions of the major protein(s) found within each protein source were calculated, and pools of free amino acids were created to represent the proportions thereof for each major dietary protein, making the general assumption that most protein substrates have been reduced to their free amino acid components through digestive processes prior to delivery to the colon. This is not an unreasonable assumption since most dietary proteins have been digested to individual amino acids or oligopeptides upon reaching the end of the ileum (31, 32).

## RESULTS

### Growth responses of *Fusobacterium* spp. to representative free amino acid pools

**Fig 1:**
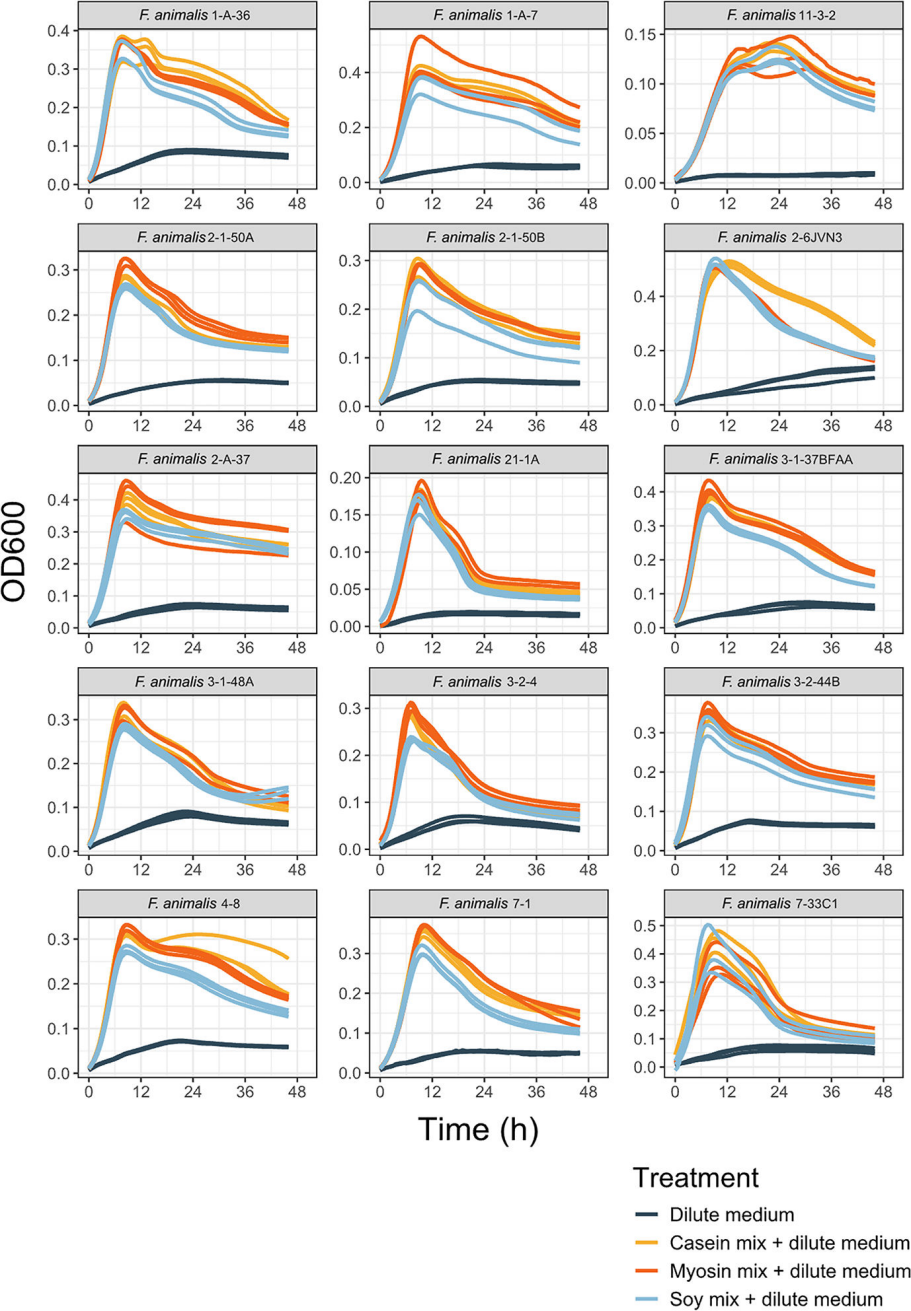

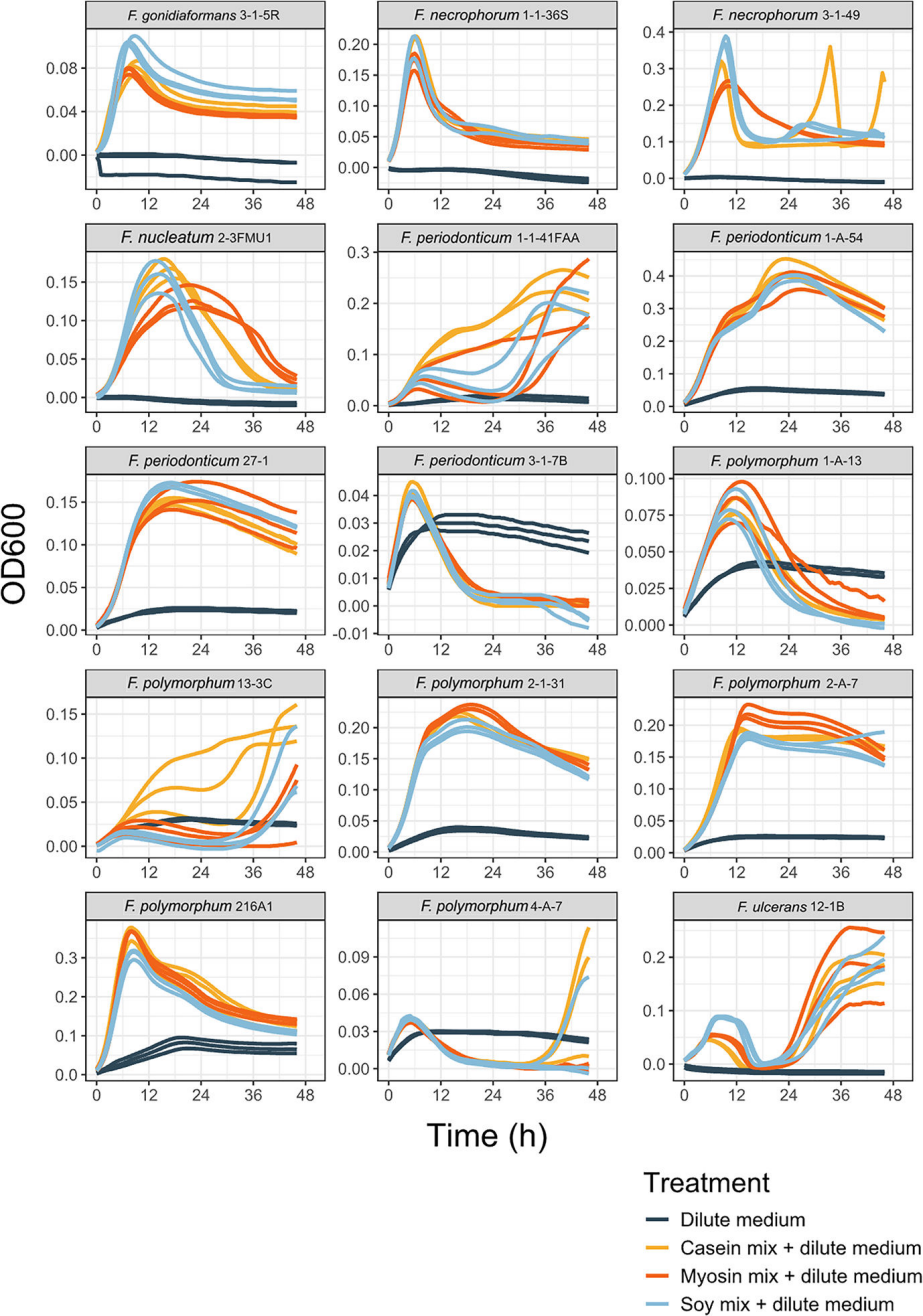

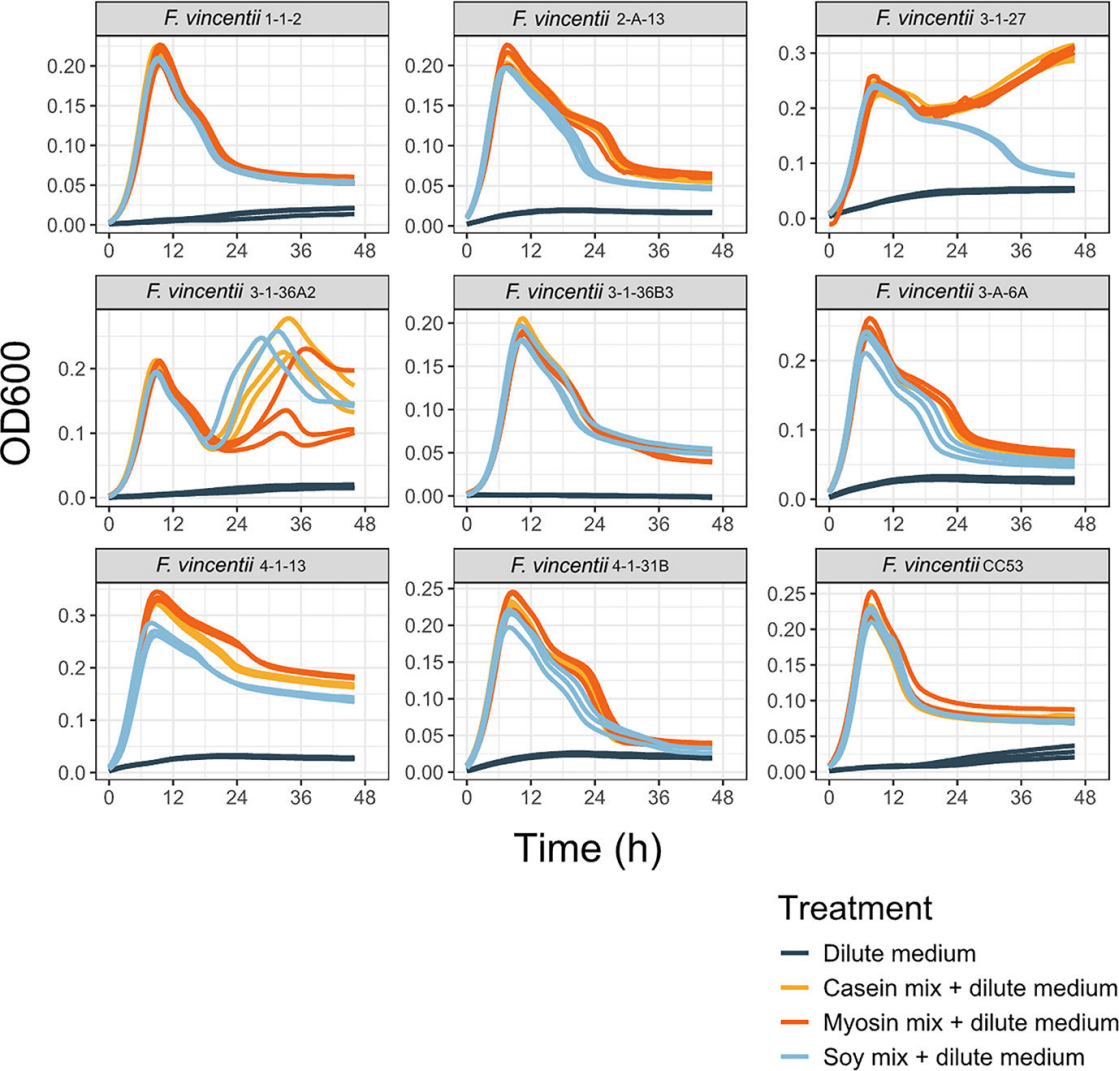
Growth curves produced by *Fusobacterium* spp. strains during the 48 hours following treatment with casein, myosin, or soy free amino acid pools (FAAPs) or control (dilute medium only). Following treatment, strains were incubated at 37°C under anaerobic, batch fermentation conditions in a spectrophotometer, which recorded optical density (OD_600_) readings at 30-minute intervals for 48 hours. OD_600_ readings were used to generate the growth curves where lines represent rolling mean over every three OD_600_ readings for each replicate.

We tested 39 strains of *Fusobacterium* spp., including 15 *F. animalis*, nine *F. vincentii*, six *F. polymorphum*, and one *F. nucleatum* for differential growth on different free amino acid pools (FAAPs) using readouts of optical density at 600 nm (OD_600_). Tested isolates were originally obtained from human oral and intestinal samples (23 from intestinal biopsies, four additional specifically from colon tumor biopsies, one each from a stomach and rectal biopsy, and 10 from oral swabs) and therefore chosen as representative and relevant strains for this study. The growth curves of the 39 *Fusobacterium* spp. strains are displayed in Fig. 1. Amino acid supplementation of *Fusobacterium* strains largely resulted in sharp death phases following initial growth, although some strains also exhibited diauxic growth (biphasic growth separated by a lag phase representative of growth when two separate nutrient sources are utilized sequentially [33]). The death phases of several strains resulted in carrying capacities that were shorter than that when grown in the dilute medium control, suggesting amino acid supplementation accelerated cell death relative to the control medium (e.g., *F. periodonticum* strain 3-1-7B). While growth curve measurements were possible for most strains, some tested isolates showed a highly aggregative phenotype that interfered with absorbance readings, and thus data for these strains were excluded from downstream analysis (Table S1).

Of the 39 *Fusobacterium* spp. strains tested, 30 strains yielded areas under the curve (AUCs) significantly higher than for controls in each of the three FAAP treatments, as determined by multiple *t*-tests (FDR-adjusted *P*-value < 0.05) (Table 1). Four additional strains yielded AUCs significantly higher than those of controls in one or two FAAP treatments; the remaining FAAP treatment(s) yielded non-statistically significant increases in AUCs (FDR-adjusted *P*-value < 0.05). Only two strains yielded AUCs significantly lower upon treatment with one or more FAAP than that of the dilute medium control. While FAAP supplementation initially allowed increased growth (the maximum growth rate achievable by the population in given conditions [r] and maximum optical density achieved [ODh]) for these two strains (compared to controls), the curves with steep death phases showed growth inhibition by FAAP treatment, starting at approximately 12 hours after supplementation.

**TABLE 1:** Average growth curve metric fold changes and *P*-values for each of the 39 *Fusobacterium* spp. strains treated with myosin, casein, and soy FAAPs or no treatment in triplicate under batch fermentation conditions^*a*^

*Fuso.* species	AUC-f Cas	AUC-f Cas p-val	AUC-f Myo	AUC-f Myo p-val	AUC-f soy	AUC-f soy p-val	r-f Cas	r-f Cas p-val	r-f Myo	r-f Myo p-val	r-f soy	r-f soy p-val	k-f Cas	k-f Cas p-val	k-f Myo	k-f Myo p-val	k-f soy	k-f soy p-val	OD-f Cas	OD-f Cas p-val	OD-f Myo	OD-f Myo p-val	OD-f soy	OD-f soy p-val
Fg	6	*	5	*	7	**													*5*	*ns*	*5*	*ns*	7	*
Fnec	7	*	6	**	*7*	*ns*													Inf	*	Inf	**	*Inf*	ns
	*37*	*ns*	40	*	40	**													*1.E+02*	*ns*	79	*	119	**
Fa	13	**	13	*	12	**	*1*	*ns*	*2*	*ns*	*2*	*ns*	14	***	14	*	13	**	14	**	14	*	13	**
	4	**	4	***	3	**	5	*	6	***	6	**	3	**	3	****	3	**	4	*	4	****	4	**
	6	**	6	*	5	*	4	**	5	***	4	**	6	***	6	*	5	*	6	**	7	*	6	*
	5	***	5	*	5	**	3	***			4	***	5	***			4	**	10	**	10	**	9	**
	4	**	4	**	4	**	7	****	7	****	7	***	3	**	4	**	3	**	5	****	6	**	5	***
	4	**	4	****	3	*	4	***	4	****	4	***	4	**	4	****	3	*	5	**	6	****	4	*
	5	***	4	**	4	**	10	***	*5*	*ns*	*9*	*ns*	3	**	2	*	2	**	4	***	4	**	4	***
	5	**	5	*	5	**	4	****	4	**	4	****	5	**	5	*	4	***	6	**	6	*	5	**
	5	****	5	**	4	****	7	****	7	****	8	****	4	***	4	***	3	***	6	****	6	**	5	****
	3	**	3	**	3	**	6	****	5	****	5	****	3	**	3	**	2	**	4	**	4	**	3	****
	3	***	3	***	3	***	6	****	5	****	*3*	*ns*	2	***	3	***	2	*	5	****	5	****	4	****
	4	**	4	**	3	*	4	***	4	****	4	****	4	**	4	**	3	**	5	**	5	**	4	**
	4	*	4	**	3	**	4	*	4	***	4	***	4	**	4	**	3	**	4	***	5	**	4	**
	5	**	5	***	4	**	4	****	4	***	4	****	4	**	5	**	3	**	6	***	6	***	5	**
	4	*	4	*	4	*	*4*	*ns*	5	*			*3*	*ns*	3	*			7	**	6	*	6	*
Fn	15	****	16	**	12	*													2.E+02	**	1.E+02	*	2.E+02	*
Fpo	*3*	*ns*	*−1*	ns	*−1*	*ns*	−3	*					*4.E+07*	*ns*					5	*	*2*	*ns*	*3*	*ns*
	*−1*	*ns*	*1*	ns	*−1*	*ns*	3	***	3	*			*−1*	*ns*	*1*	*ns*			2	*	*2*	*ns*	2	*
	6	**	6	****	6	**	1	**	1	***	1	***	6	**	6	****	6	**	6	**	6	****	6	**
	3	**	3	**	3	**	*3*	*ns*	5	**	5	**	3	**	3	**	3	**	5	***	5	**	4	***
	7	***	7	**	6	**	1	**	1	**	*1*	*ns*	7	****	8	**	7	**	7	****	9	**	7	**
	*−1*	ns	−2	*****	*−2*	*ns*	*−3*	*ns*					*3*	*ns*					*3*	*ns*	1	*	*2*	*ns*
Fv	8	****	8	**	8	***	*5*	*ns*	*5*	*ns*	*6*	*ns*	*4*	*ns*	*4*	*ns*	*4*	*ns*	12	****	12	**	11	***
	7	**	7	**	6	***	4	****	4	**	4	***	6	**	6	**	5	***	11	**	11	**	10	****
	5	**	5	***	3	****	4	**			7	***	5	**			3	****	6	**	6	***	5	****
	14	*	11	*	14	****													14	*	13	**	15	*
	3.E+02	**	3.E+02	**	3.E+02	**													2.E+02	*	1.E+02	***	141	**
	4	***	5	**	4	*	5	****	5	***	5	****	4	***	4	***	4	*	8	****	8	**	7	**
	8	***	8	***	7	**	5	****	4	****	5	**	7	***	8	***	6	***	10	***	10	**	9	**
	5	***	5	**	4	*	4	****	4	****			5	****	5	**			9	***	10	**	9	**
	7	***	7	**	6	***	23	****	20	****	23	****	3	***	3	**	3	**	8	***	8	**	8	***
Fp	13	*	*7*	ns	*7*	*ns*	−3	**	*−2*	*ns*	−3	*	17	*	*3.E+07*	*ns*	*20*	*ns*	14	*	*13*	*ns*	12	*
	7	**	7	**	7	****	−1	**	−1	**	−2	**	8	**	8	**	7	****	8	**	7	**	8	***
	5	**	6	*	6	****	2	**	*2*	*ns*	2	**	6	**	6	*	6	****	6	***	6	*	7	****
	−2	**	−2	*	−3	**													1	*	1	*	1	*
Fu	5	*	*6*	*ns*	6	**													Inf	*	*Inf*	*ns*	Inf	*

^*a*^ Fa: *F. animalis*; Fg: *F. gonidiaformans*; Fnec: *F. necrophorum*; Fn: *F. nucleatum*; Fpe: *F. periodonticum*; Fpo: *F. polymorphum*; Fu: *F. ulcerans*; Fv: *F. vincentii*; Cas: casein; Myo: myosin; AUC: area under the curve; AUC-f: fold change in AUC; r: maximum growth rate; r-f: fold change in r; k: carrying capacity; k-f: fold change in k; ODh: mean of three highest optical density readings; ODh-f: fold change in ODh; p-val: false discovery rate (FDR)-adjusted *P*-value; ns: not significant; and Inf: infinity. Values of AUC-f, r-f, k-f, and ODh-f represent the fold change between treatments, where a positive value indicates cases where FAAP-treatment resulted in a higher value than the control, while a negative value indicates cases where the no-treatment control resulted in a higher value than the FAAP-treatment. Inf was produced when the denominator was 0 when calculating the fold change. *P*-values were FDR-corrected using the Benjamini–Hochberg method and are indicated with asterisks: **** = *P*-value < 0.0001, *** = *P*-value < 0.001, ** = *P*-value < 0.01, * = *P*-value < 0.05. Underlining indicates cases where the value of the FAAP-treated condition was larger than the value of the control condition, while shaded cells indicate cases where the value of the control condition was larger than the value of the FAAP-treated condition. Fold change values that were not significant are italicized. Empty cells indicate that the growth curves for the given condition(s) for that strain were not well-fitted.

Logistic regression estimating growth curve characteristics (r and carrying capacity [k]) were well-fitted for 24 out of 39 strains tested in all replicates and all conditions. Relative to the dilute medium controls, and as expected, FAAP addition significantly increased the number of generations/time (k values) for most strains with well-fitted growth curves (see Table 1; FDR-adjusted *P*-values < 0.05). However, while FAAP addition significantly increased the maximum growth rates achieved (r values) for most well-fitted growth curves compared to controls, FAAP addition also significantly decreased the r value(s) for several growth curves compared to the dilute medium control (FDR-adjusted *P*-values < 0.05). As expected, the maximum optical density achieved (ODh) was significantly higher than that of controls for most strains (casein: 36/39; myosin: 34/39; soy: 36/39; FDR-adjusted *P*-values < 0.05).

**Fig 2:**
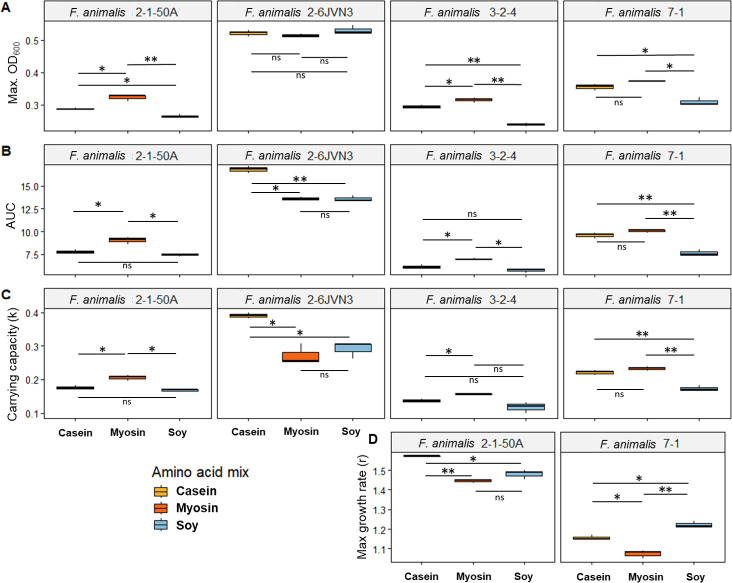
Boxplots of growth curve metrics for select *Fusobacterium animalis* strains following treatment with casein, myosin, or soy free amino acid pools (FAAPs) where all FAAP treatments were significantly different from controls. (A) Maximum optical density at 600 nm wavelength light (Max. OD_600_) reached for *F. animalis* strains 2-1-50A, 2-6JVN3, 3-2-4, and 7-1. (B) Areas under the curve (AUC) reached for *F. animalis* strains 2-1-50A, 2-6JVN3, 3-2-4, and 7-1. (C) Carrying capacity (k) reached for *F. animalis* strains 2-1-50A, 2-6JVN3, 3-2-4, and 7-1. (D) Maximum growth rate (r) reached for *F. animalis* strains 2-1-50A and 7-1. *t*-tests with Benjamini–Hochberg false-discovery rate corrections were conducted in R using the package rstatix. Corrected *P*-values are shown by asterisks as follows: **** = *P*-value < 0.0001; *** = *P*-value < 0.001; ** = *P*-value < 0.01; * = *P*-value < 0.05; ns = not significant.

Comparing growth curve metrics among isolates that significantly differed from controls for all three FAAP conditions, we found that FAAP treatment influence on fusobacterial growth metrics was inconsistent between strains. For example, while several strains exhibited significantly increased overall growth (AUCs) with myosin FAAP treatment compared to both casein and soy FAAPs (e.g., *F. animalis* strain 2-1-50A), others exhibited significantly increased overall growth with both myosin and casein FAAP treatments compared to soy FAAP (e.g., *F. animalis* strain 7-1) (Fig. 2; Fig. S1; FDR-adjusted *P*-values < 0.05). These strain-specific differences in overall growth for each FAAP treatment largely translated to the carrying capacity (sustainable cellular density; k value): (i) significantly increased carrying capacity with myosin FAAP treatment compared to both casein and soy FAAP (e.g., *F. animalis* strain 2-1-50A); (ii) significantly increased carrying capacity with both myosin and casein FAAP treatments compared to soy FAAP (e.g., *F. animalis* strain 7-1); and (iii) significantly increased carrying capacity with casein FAAP treatment compared to both myosin and soy FAAPs (e.g., *F. animalis* strain 26JVN3) (Fig. 2; Fig. S2; FDR-adjusted *P*-values < 0.05). The maximum optical density (ODh) reached with each FAAP treatment likewise varied largely between strains: some strains reached a significantly higher cell density with myosin and casein FAAP compared to soy (e.g., *F. animalis* strain 7-1), while others grew to a significantly higher cell density in myosin FAAP alone (e.g., *F. animalis* strain 3-2-4) (Fig. 2; Fig. S3; FDR-adjusted *P*-values < 0.05). The trends in the maximum achieved growth rate (r values) for fusobacterial strains were largely unique compared to that of the other three growth curve metrics. For example, the maximum growth rate of the myosin FAAP-treated *F. animalis* strain 7-1 was significantly lower than that for casein and soy treatments, despite myosin FAAP treatment yielding the highest overall growth, carrying capacity, and maximum cellular density achieved by strain 7-1 (Fig. 2; Fig. S4; FDR-adjusted *P*-values < 0.05).

### Amino acid utilization specificities of *F. nucleatum* strains

**Fig 3:**
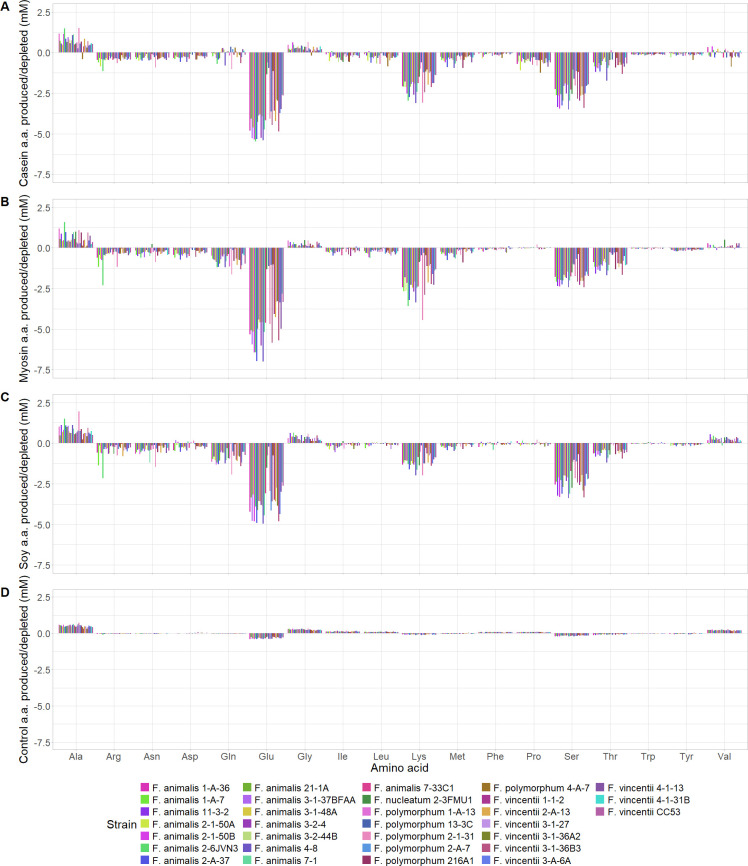
Bar plot visualization of amino acid depletion and production by each *Fusobacterium* spp. strain relative to the sterile medium in casein free amino acid pool (FAAP), myosin FAAP, soy FAAP, and control conditions at the time point of supernatant collection. Amino acid (a.a.) three letter codes are alphabetically listed on the *X*-axis. Amino acids were detected and quantified in strain monoculture supernatants and sterile controls using 1D ^1^H nuclear magnetic resonance spectroscopy (NMR) (*N* = 1 FAAP treatment per strain). *Fusobacterium* spp. strains are arranged on the *X*-axis alphabetically.

To assess the amino acid preference of *Fusobacterium* spp. strains during log phase growth, amino acid concentrations in culture supernatants for 31 strains across four species (*F. animalis*, F. *nucleatum*, *F. polymorphum*, and *F. vincentii*; Table 2) and sterile broth controls were measured using 1D proton nuclear magnetic resonance (NMR) on samples taken from the first time point for each representative strain where a maximum OD_600nm_ was reached in at least one condition (casein, myosin, or soy FAAP treatment) (Table S2). To accurately assess metabolite concentrations by NMR, sample pH values were measured immediately following scans (Table S3). All amino acids but cysteine (Cys) and histidine (His) were detectable, possibly due to low concentrations in FAAPs. Trends seen in the amino acid profiles of many tested *Fusobacterium* strains included >30% depletion compared to the sterile medium of glutamate, lysine, serine, and threonine (Glu, Lys, Ser, and Thr, respectively) (Fig. 3; Table S4). *Fusobacterium* strains were less consistent in their depletion of glutamine, methionine, tryptophan, and tyrosine (Gln, Met, Trp, and Tyr, respectively) and rarely depleted arginine, asparagine, and aspartate (Arg, Asn, and Asp). Many *Fusobacterium* strain supernatants yielded higher concentrations of alanine (Ala) and valine (Val) compared to controls. However, the degree of each amino acid’s depletion varied both between and within strains when considering different FAAP treatments.

**TABLE 2:** *Fusobacterium* spp. strains used in free amino acid pool growth curve assays, with details of associated patient metadata and original citation^*a*^

Species	Strain	Disease status of the host	Sampling site from which the strain was isolated	Assessed for amino acid utilization	Citation
*F. gonidiaformans*	3-1-5R	Active ulcerative colitis	Intestinal biopsy	−	Strauss et al. (34)
*F. necrophorum*	1-1-36S	Active Crohn’s	Terminal ileum biopsy	−	
	3-1-49	Active ulcerative colitis	Cecum biopsy	−	
*F. animalis*	2-1-50A	Crohn’s: in remission	Distal colon biopsy	+	
	3-2-4	Crohn’s: in remission	Cecum biopsy	+	
	1 A-7	Active Crohn’s	Mouth swab	+	
	1 A-36	Active Crohn’s	Mouth swab	+	
	2 A-37	Indeterminate colitis	Mouth swab	+	
	3-1-37BFAA	Indeterminate colitis	Terminal ileum biopsy	+	
	3-2-44B	Crohn’s: in remission	Descending colon biopsy	+	
	3-1-48A	Indeterminate colitis	Distal ileum biopsy	+	
	2-1-50B	Crohn’s: in remission	Descending colon biopsy	+	
	2-6JVN3	Colorectal cancer	Colon tumor biopsy	+	Cochrane (35)
	4–8	Healthy, colon cancer screen	Sigmoid colon biopsy	+	Strauss et al. (34)
	7-1	Active Crohn’s	Sigmoid colon biopsy	+	
	11-3-2	Healthy, colon cancer screen	Nonspecific colon biopsy	+	
	22-6A	Healthy, colon cancer screen	Sigmoid colon biopsy	+	
	7-33C1	Colorectal cancer	Colon tumor biopsy	+	Cochrane (35)
*F. nucleatum*	2-3FMU1	Colorectal cancer	Colon tumor biopsy	+	
*F. polymorphum*	13-3C	Crohn’s, in remission	Sigmoid colon biopsy	+	Strauss et al. (34)
	2 A-7	Active Crohn’s	Mouth swab	+	
	4 A-7	Active Crohn’s	Mouth swab	+	
	1 A-13	Healthy, colon cancer screen	Mouth swab	+	
	2-1-31	Active Crohn’s	Ascending colon biopsy	+	
	216A1	Oral cancer	Oral tumor brush	+	This work
*F. vincentii*	4-1-13	Healthy, colon cancer screen	Ascending colon biospy	+	Strauss et al. (34)
	2 A-13	Healthy, colon cancer screen	Mouth swab	+	
	3-1-27	Healthy, colon cancer screen	Rectum biopsy	+	
	4-1-31B	Active Crohn’s	Ascending colon biopsy	+	
	3-1-36B3	Active Crohn’s	Ascending colon biopsy	+	
	3-1-36A2	Active Crohn’s	Ascending colon biopsy	+	
	1-1-2	Healthy, colon cancer screen	Nonspecific colon (biopsy site not recorded)	+	Strauss (36)
	3 A-6A	Healthy donor	Oral swab	+	Strauss et al. (34)
	CC53	Colorectal cancer	Colon tumor biopsy	+	Cochrane (35)
*F. periodonticum*	27-1	Healthy, colon cancer screen	Terminal ileum biopsy	−	Strauss et al*.* (34)
	3-1-7B	Active Crohn’s	Sigmoid colon biopsy	−	
	1-1-41FAA	Healthy, colon cancer screen	Stomach biopsy	−	
	1 A-54	Active Crohn’s	Oral swab	−	
*F. ulcerans*	12-1B	Active Crohn’s	Colon biopsy	−	

^*a*^ Abbreviations: +, assessed for amino acid depletion patterns; –, not assessed for amino acid depletion patterns.

### Fusobacterium spp. strain heterogeneity in free amino acid pool-linked metabolism

Strain-level heterogeneity in fusobacterial utilization of amino acids within the species was evaluated using k-means clustering (37) based on either growth curve metrics (fold changes in AUC and ODh in response to the three FAAP treatments) or amino acid depletion patterns in all conditions. Strains that clustered together both by growth metrics and amino acid utilization were considered to have similar metabolic strategies. However, *Fusobacterium* spp. demonstrated a high degree of intraspecies variation in FAAP treatment-driven growth curve metrics and amino acid depletion profiles (Table 3). Furthermore, there was also a high degree of inter- and intraspecies variation within and between strains of *F. animalis*, *F. polymorphum*, and *F. vincentii*. In contrast, *F. nucleatum* strain 2-3FMU1 shared the same amino acid depletion and growth curve metrics as several strains of *F. vincentii*.

**TABLE 3:** Heterogeneity in metabolism of amino acids among 31 strains of *Fusobacterium* spp., as determined by k-means clustering based on strain growth curve metrics or amino acid utilization profiles^*a*^

*Fusobacterium* species	Strain	Amino acid utilization cluster	Growth curve metrics cluster
*F. animalis*	2 A-37	1	2
	7-1	1	3
	4-8	1	5
	2-6JVN3	2	2
	1 A-7	3	2
	2-1-50A	3	3
	3-1-48A	3	3
	1 A-36	3	5
	3-1-37BFAA	3	5
	3-2-44B	3	5
	7-33C1	3	5
	3-2-4	3	6
	2-1-50B	6	3
	11-3-2	6	4
	21-1A	6	4
*F. nucleatum*	2-3FMU1	5	4
*F. polymorphum*	216A1	1	3
	2-1-31	2	6
	1 A-13	4	1
	13-3C	4	1
	4 A-7	4	1
	2 A-7	4	6
*F. vincentii*	4-1-13	3	3
	3-1-27	5	3
	1-1-2	5	4
	3 A-6A	5	4
	4-1-31B	5	4
	CC53	5	4
	3-1-36A2	5	6
	2 A-13	6	4
	3-1-36B3	6	4

^*a*^ Growth curve metrics, free amino acid pool (FAAP)-induced fold changes in area under the curve, and mean highest achieved OD readings were used to group strains into six clusters. Independently, amino acid utilization profiles were used to group strains into six clusters. The number of clusters in each case was determined using the elbow method by plotting the within-cluster sum of squares against the number of clusters (up to 25 clusters).

Despite this apparent heterogeneity, some metabolically similar groupings (≥2 strains) within fusobacterial species emerged: three distinct groups within *F. animalis*, one in *F. polymorphum*, and two in *F. vincentii*. However, interspecies metabolically-similar groups also became apparent, including groups overlapping in *F. animalis* (strains 11-3-2 and 21-1A) and *F. vincentii* (strains 2 A-13 and 3-1-36B3). Other groups within species shared similar clusters with single strains in other species—for example, generally *F. vincentii* strains clustered with *F. animalis* strains 2-1-50A and 3-1-48A. Yet, even within these metabolically-similar groups, there were inconsistencies in growth curve shapes. For example, *F. animalis* strains 11-3-2 and 21-1A clustered by both growth curve metrics and amino acid use, although strain 11-3-2 exhibited diauxic growth in response to FAAP treatments, whereas strain 21-1A yielded a more distinctive death phase. Strains within each species, while clustering together in terms of their growth curves, exhibited different patterns of amino acid use, although the reverse situation was also found. For example, for *F. animalis*, two clusters calculated using growth curve metrics similarity matrices each contained strains that clustered together differently according to their amino acid depletion patterns, and three clusters calculated from similarity matrices from amino acid depletion data contained strains that clustered differently according to their growth curves.

**Fig 4:**
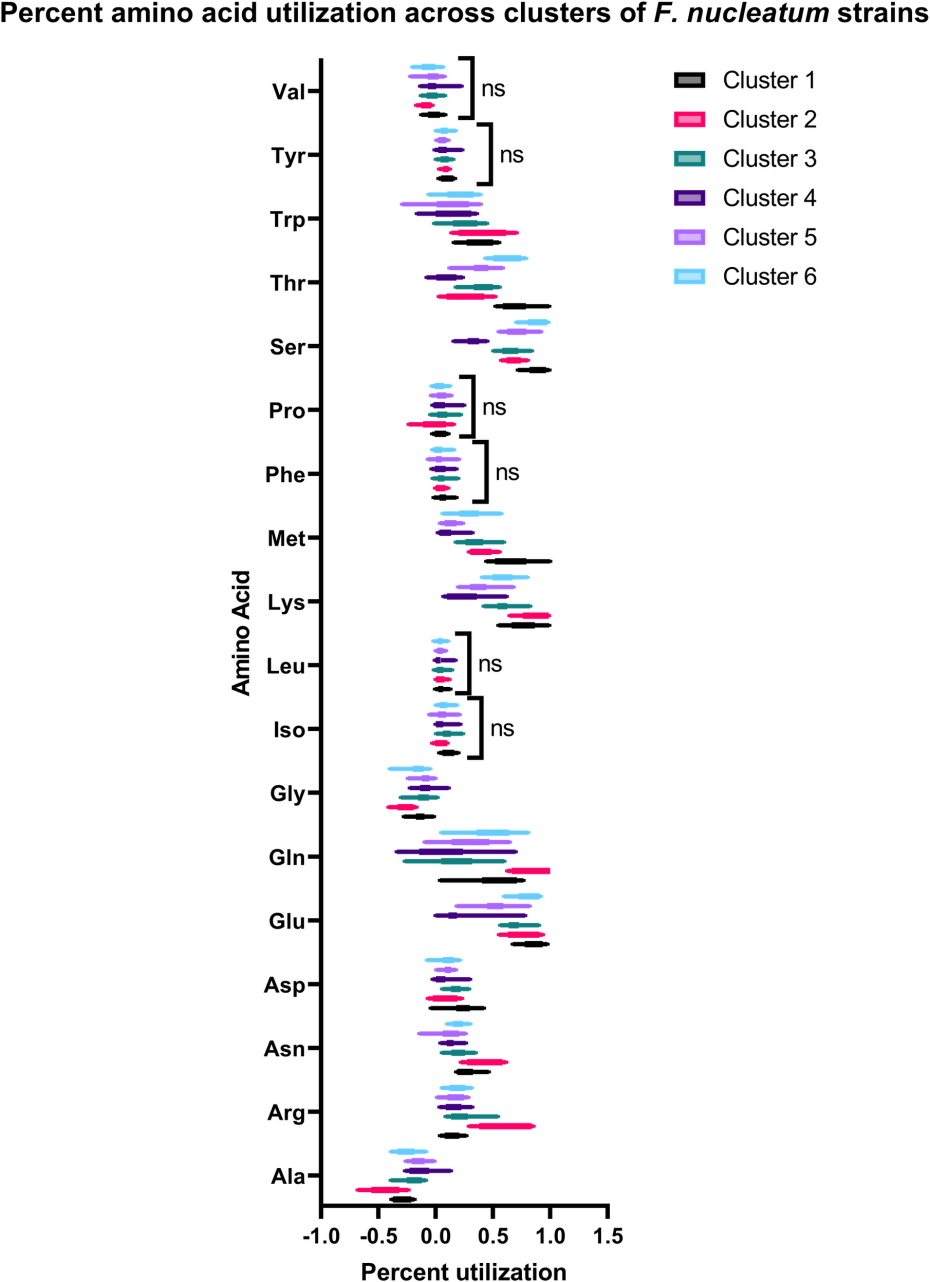
Boxplots of amino acid percent utilization in all free amino acid pool (FAAP) treatment conditions for *Fusobacterium* spp. strain groupings determined by k-means clustering of amino acid utilization profiles. Contribution of each amino acid utilization profile to k-means clustering was determined by a two-way ANOVA test comparing each cluster’s strain amino acid utilization percentages for each FAAP treatment between all clusters, followed by Tukey’s honest significant difference *post-hoc* tests. Amino acids whose utilization percentage profiles do not significantly differ between any of the *Fusobacterium* spp. strain clusters are denoted “ns” for “not significant.” All other amino acid utilization percentage profiles were significantly different between at least one pair of strain clusters (see Table S5).

To determine which amino acid depletion trends contributed to the observed intraspecies heterogeneity within *Fusobacterium* spp., percent depletion for each amino acid for each strain was pooled across all three FAAP treatments. *Fusobacterium* strains that were determined similar by k-means clustering were grouped, and differences in mean percent utilization across all 18 amino acids detected between each cluster were evaluated using two-way analysis of variance (ANOVA), followed by Tukey’s honest significant difference (HSD) *post-hoc* tests. Depletion of only six of 18 detected amino acids was determined to not be significantly different between at least one cluster pair within the 31 fusobacterial strains profiled: isoleucine, leucine, phenylalanine, proline (Iso, Leu, Phe, and Pro), Tyr, and Val (Fig. 4); all other amino acid percent utilizations were significantly different between at least two fusobacterial strain clusters (Table S5). This suggests that *Fusobacterium* spp. metabolize a wide range of amino acids, and metabolic “preference” (e.g., degree and order of use) varies at the strain level (Table S4).

Finally, we were interested to determine whether *F. animalis* strains that identified with the recently described clade C2, associated with enrichment in CRC (38), might also cluster with respect to their growth in response to FAAPs. We assessed strains for which associated genomes were available (Table S5). All of the tested *F. animalis* strains fell into clade C2 (Fig. S5), suggesting that *F. animalis* taxonomic classification still does not explain differences in amino acid preference in these isolates.

## DISCUSSION

In this study, 39 *Fusobacterium* spp. strains originally cultivated from human oral and intestinal samples were treated with FAAPs representative of different dietary proteins. Following the initial peak in average OD_600_ readings, some *Fusobacterium* strains entered a second lag and growth phase, indicative of diauxic growth (e.g., *F. animalis* strain 11-3-2). In conditions with more than one nutrient resource, diauxic growth arises when a bacterial strain preferentially utilizes one resource to complete depletion, whereafter the cells switch their metabolic strategy to utilize a second resource, requiring a period of minimal growth to express the second set of genes required (33). Those strains that did not exhibit diauxic growth entered the death phase following the initial peak average OD_600_, suggesting swift cell death following complete depletion of preferred nutrients and/or accumulation of waste products (e.g., *F. polymorphum* strain 4 A-7). Waste products from proteolytic fermentation by fusobacteria can include organic acids such as acetate, butyrate, propionate, and lactate (39). Other strains (e.g., *F. vincentii* strain 3-1-36A2) displayed irregularities during or instead of a death phase, such as inconsistent increases in OD_600_ following the initial peak in growth. Inconsistencies in OD_600_ readings aligned with visible aggregation within wells (some *Fusobacterium* spp. are known to be highly aggregative [40]). However, the cause of this aggregation cannot be attributed to nutrient starvation or proteolysis waste product toxicity without further investigation of strain transcriptional responses during FAAP treatment (40).

A high degree of heterogeneity was observed between and within *Fusobacterium* species. When comparing casein, myosin, and soy FAAP treatment effects on the AUC, carrying capacity, maximum growth rate, and ODh of each strain, only some differences in metrics between treatments were significant. Of those significant growth metric differences, there were no consistent trends within the *Fusobacterium* genus nor within individual *Fusobacterium* species. The most frequently observed significant effect was an increase in AUC, carrying capacity, and maximum optical density reached (ODh) upon myosin or myosin and casein FAAP treatment(s) compared to soy FAAP treatment. However, some strains exhibited different growth metrics, such as *F. animalis* strain 2-6JVN3 yielding higher AUC and carrying capacity with casein FAAP treatment alone compared to myosin and soy FAAP treatments. The inconsistencies in direction, magnitude, and significance of growth metric differences between strains are suggestive of strain-specific variation in amino acid usage and rates of metabolism. The present study indicates that Glu, Lys, Ser, and Thr are common *Fusobacterium* spp. metabolic substrates and that some strains also utilize Gln, Met, Trp, and Tyr, but rarely Arg, Asn, and Asp. This aligns closely with previously established *Fusobacterium* spp. amino acid utilization patterns, which include Asp, Cys, Glu, Gln, His, Lys, Met, Ser, and Thr as primary metabolic substrates, and occasional utilization of Trp and Tyr (25, 39, 41). Although inconsistent, the trend in preference for the myosin and/or casein FAAP(s) correlates with the observed and previously established amino acid substrate preferences of fusobacteria and the proportions of relevant amino acids in each FAAP.

The amino acid substrate preference of *Fusobacterium* spp. strains during the logarithmic phase was investigated by collecting supernatants for amino acid profiling either at or just before the first peak in growth was reached. Although tested strains were mostly consistent in their amino acid preferences, strain-specific exceptions were observed. For example, although Glu was a common metabolic substrate for most tested strains (>30% utilization in all conditions), four strains utilized <30% Glu in all three FAAP treatments: *F. polymorphum* strains 1 A-13, 4 A-7, and 13-3C, and *F. nucleatum* strain 2-3FMU1. *F. polymorphum* strains 1 A-13, 4 A-7, and 13-3C and *F. nucleatum* strain 2-3FMU1 displayed typical preferences for amino acids, as previously reported (25, 39, 41); however, *F. polymorphum* strain 4 A-7′s utilization of >30% Arg in the casein FAAP treatment condition was unusual (25, 39, 41).

Nine distinct amino acid profile and growth curve metric cluster combinations were determined within *F. animalis* strains; four within *F. polymorphum* strains and five within *F. vincentii* strains. Furthermore, several amino acid profile and growth curve metric cluster combinations were shared among strains of distinct species, suggesting that species delineation is not predictive of a given strain’s amino acid metabolic preferences. Dzink and Socransky (41) previously suggested inconsistencies between *Fusobacterium* spp. usage of amino acids using a small number of strains. Our work significantly expands on these findings and more clearly demonstrates that differences in amino acid preferences are highly heterogenous among fusobacterial strains within and across several species and may not be easily explained using existing phylogenetic frameworks.

The casein, myosin, and soy FAAPs used in this experiment were designed to represent the typical amino acid proportions within the most abundant protein type in three dietary protein sources: bovine milk, beef, and soy (42–44). The proportions of amino acids within the FAAPs varied by as much as ~9% of the total amino acid content (Pro in casein FAAP compared to myosin FAAP) and as little as <0.2% (Cys in myosin FAAP compared to casein FAAP). Of the amino acids commonly used by *Fusobacterium* strains within this experiment (Glu, Lys, Ser, Thr, Gln, Met, Trp, and Tyr), proportions varied by ~6% and ~0.15% between FAAPs. Despite these relatively small differences in amino acid proportions between the three FAAPs, there was a significant influence on growth curve metrics for several strains, including *F. animalis* strain 7-1, originally isolated from a colonic biopsy of a region of active inflammation in a Crohn’s disease patient (34). Strain 7-1 has been shown to promote intestinal tumorigenesis within a *Apc^Min/+^* mouse model (13). Free amino acids can reach the colon following transit through the small intestine, as shown in both gnotobiotic and conventional mouse models (24). While animal- and plant-based diets have both been shown to provide all amino acids (26, 45–47), individual foods can vary in amino acid composition (27–30). Thus, individual dietary proteins, if digested into free amino acids that reach the colon, may influence the growth of potentially pathogenic strains of *Fusobacterium* spp..

In summary, this study has demonstrated that varying proportions of amino acids reflective of dietary proteins can differentially influence the growth of *Fusobacterium* spp., a group of bacteria containing strains with known pathogenic tendencies in the gut, serving as justification for further characterization of the effects of dietary protein sources on potential pathogens within the context of intestinal disease.

## MATERIALS AND METHODS

### *Fusobacterium* spp. strain isolation, selection, and growth conditions

One strain of *Fusobacterium* spp., *F. polymorphum* strain 216A1, was newly isolated for this work. Oral cancer cytobrush swabs were collected in 15-mL conical tubes and stored at −80°C. Samples were shipped on dry ice to the University of Guelph (Guelph, Canada) for culturing under University of Guelph REB#19-11-013. Cytobrush sample conicals were transferred to an anaerobic chamber (AS-580, Anaerobe Systems, Morgan Hill, CA, USA) for culturing work. Brain heart infusion broth (BHI) was previously reduced in anaerobic conditions (10% H_2_, 10% CO_2_, and N_2_ balance) >18 hours prior to 5 mL BHI addition to cytobrush sample conical tubes. Cytobrush conical tubes were then vortexed on maximum speed for 1 minute. Aliquots of the resulting cell suspension were plated on fastidious anaerobe agar (Neogen, Lansing, MI, USA) supplemented with 5% (vol/vol) defibrinated sheep’s blood (Hemostat Laboratories, Dixon, CA, USA) (FAA), and on FAA supplemented with 3 µg/mL josamycin, 4 µg/mL vancomycin, and 1 µg/mL norfloxacin (JVN). Inoculated FAA and JVN plates were incubated 72 hours anaerobically at 37°C prior to identification of isolates by 16S rRNA gene variable regions 3–6 (V3–V6) Sanger sequencing and comparison against the NCBI sequence database were done as previously described (48).

The human oral and intestinal sample-derived strains of *Fusobacterium* spp. used in the current study and the donor patient metadata can be found in Table 2. Species identity was confirmed by 16S rRNA gene variable regions 3–6 (V3–V6) Sanger sequencing with comparison against the NCBI sequence database. The phylogenetic tree of 16S rRNA gene V3–V6 sequences for fusobacterial strains used was built using the online tool Phylogeny.fr (49). Strains were inoculated from frozen stocks onto FAA and incubated 48–72 hours at 37°C in an anaerobe chamber under anaerobic conditions. About 1 µL of biomass from each strain grown on FAA was transferred to previously reduced (>18 hours) modified peptone–yeast–glucose (mPYG) broth (5 g Bacto Peptone (BD Biosciences, Mississauga, ON, Canada), 5 g Bacto Yeast Extract (Organotechnie, La Courneuve, France), 2.5 g glucose (ThermoFisher, Waltham, MA, USA), filled to 500 mL deionized H_2_O for autoclaving at 121°C and 15 p.s.i. for 30 minutes; post-autoclave, 0.25 g L-cysteine-HCl-H_2_O (ThermoFisher), 0.25 g (NH_4_)_2_SO_4_ (BDH Belt, Conestoga, PA, USA), 16.7 µg menadione (Sigma-Aldrich), 200 mg NaHCO_3_ (ThermoFisher), 40 mg NaCl (ThermoFisher), 20 mg K_2_HPO_4_ (ThermoFisher), 20 mg KH_2_PO_4_ (ThermoFisher), 4 mg CaCl_2_ (Sigma-Aldrich), 4 mg MgSO_4_ (Sigma-Aldrich), 5 mL volatile fatty acids and vitamin solution (10 mg pyridoxine (Sigma-Aldrich), 5 mg p-aminobenzoic acid (PureBulk, Roseburg, OR, USA), 5 mg nicotinic acid (Sigma-Aldrich), 5 mg pantothenic acid (PureBulk), 5 mg riboflavin (Sigma-Aldrich), 5 mg thiamine–HCl (Sigma-Aldrich), 2 mg biotin (PureBulk), 2 mg folic acid (Sigma-Aldrich), 0.1 mg vitamin B12 (Sigma-Aldrich), all dissolved in 1 L deionized H_2_O, pH-corrected to 7.0 with 2 N NaOH and 1 M HCl as required, then filter-sterilized with 0.2-µm polyether sulfone (PES) filter, and stored at 4°C protected from light) were added; post-addition, the medium was filter-sterilized with a 0.2-µm PES filter and stored at 4°C protected from light). Strains were allowed to grow for 24 hours anaerobically at 37°C prior to dilution for the commencement of growth curve measurements.

### Free amino acid pool design and composition

Free amino acid pools (FAAPs) were based on three dietary sources of protein: beef, bovine milk, and soy. The most abundant protein was selected from each: myosin from beef (43), casein from bovine milk (42), and glycinin and β-conglycinin in soy (44). As each protein has multiple isoforms or subunits, all isoforms were considered when calculating amino acid proportions (42, 50, 51). The amino acid sequence from each protein isoform or subunit was taken from UniProt (https://www.uniprot.org/; Table 4). Amino acid proportions were calculated for each isoform and subunit using the online Protein Calculator tool (52). Weighted averages for each amino acid in each protein source [myosin, casein, and soy (β-con)glycinin (soy)] were calculated based on abundances of each isoform within the dietary protein source (42, 44, 51) (Table 5). Individual amino acids were sourced from Sigma-Aldrich (St. Louis, MO, USA). FAAP stocks were made up to 200 mM total amino acid concentration in deionized water, and 2 N NaOH was added dropwise until all amino acids were fully dissolved, and then solutions were filter-sterilized through a Corning 0.22-µm Disposable Bottle Top Filter (ThermoFisher Scientific, Waltham, MA, USA). Thereafter, FAAP treatments were created by combining FAAP stocks to a final concentration of 50 mM total amino acid concentration with 10% (vol/vol) mPYG, which were then pH-corrected to 7.0 with 1 M HCl and filter-sterilized as before. FAAP treatments were stored at 4°C, protected from light.

**TABLE 4:** Dietary protein representative free amino acid pools (FAAPs), the major protein(s) found therein, and gene product isoforms from which amino acid proportions were analyzed in FAAP generation^*a*^

Dietary protein FAAP	Dietary protein source	Gene product isoform	UniProt entry ID	Protein name
Myosin	Beef skeletal myosin	MyHC-slow	Q9BE39 · MYH7_BOVIN	Myosin heavy chain slow isoform (MyHC-slow)
		MyHC-2a	Q9BE41 · MYH2_BOVIN	Myosin heavy chain 2 a (MyHC-2a)
		MyHC-2x	Q9BE40 · MYH1_BOVIN	Myosin heavy chain 2 x (MyHC-2x)
Casein	Bovine milk casein	as1	P02662 · CASA1_BOVIN	Alpha-S1-casein
		as2	P02663 · CASA2_BOVIN	Alpha-S2-casein
		beta	P02666 · CASB_BOVIN	Beta-casein
		kappa	P02668 · CASK_BOVIN	Kappa-casein
Soy	Soy β-conglycinin	glcb1	P25974 · GLCB1_SOYBN	Beta-conglycinin beta subunit 1
		glcap	P11827 · GLCAP_SOYBN	Beta-conglycinin alpha' subunit
		glca1	P0DO16 · GLCA1_SOYBN	Beta-conglycinin alpha subunit 1
		glca2	P0DO15 · GLCA2_SOYBN	Beta-conglycinin alpha subunit 2
		glcb2	F7J077 · GLCB2_SOYBN	Beta-conglycinin beta subunit 2
	Soy glycinin	glyg1	P04776 · GLYG1_SOYBN	Glycinin G1
		glyg2	P04405 · GLYG2_SOYBN	Glycinin G2
		glyg3	P11828 · GLYG3_SOYBN	Glycinin G3
		glyg4	P02858 · GLYG4_SOYBN	Glycinin G4
		glyg5	P04347 · GLYG5_SOYBN	Glycinin G5

^*a*^ The UniProt entry identity (ID) for each isoform is given along with the resulting protein name from the respective UniProt entry page.

**TABLE 5:** Percentage of each amino acid relative to total amino acids in each of casein-, myosin-, and soy-derived free amino acid pools (FAAPs) used in the *Fusobacterium* spp. growth curve assay

Amino acid	Casein FAAP (% total)	Myosin FAAP (% total)	Soy FAAP (% total)
Alanine (Ala)	5.3	8.7	5.1
Cysteine (Cys)	0.6	0.8	1.3
Aspartate (Asp)	2.4	5.1	4.4
Glutamate (Glu)	9.8	13.5	9.6
Phenylalanine (Phe)	3.9	3.0	4.9
Glycine (Gly)	2.8	3.7	6.0
Histidine (His)	2.1	1.9	1.9
Isoleucine (Ile)	5.5	5.0	4.8
Lysine (Lys)	6.8	10.7	4.9
Leucine (Leu)	10.1	10.5	8.5
Methionine (Met)	2.8	2.6	0.9
Asparagine (Asn)	3.6	4.2	7.0
Proline (Pro)	10.6	1.7	6.3
Glutamine (Gln)	7.6	6.6	8.9
Arginine (Arg)	2.4	5.5	6.6
Serine (Ser)	7.4	4.8	7.4
Threonine (Thr)	4.5	5.0	3.1
Valine (Val)	7.4	4.2	5.5
Tryptophan (Trp)	0.7	0.5	0.6
Tyrosine (Tyr)	3.8	2.1	2.5

### *Fusobacterium* spp. strain growth curves

*Fusobacterium* spp. growth curves were prepared anaerobically. *Fusobacterium* spp. strains were cultured overnight in mPYG broth and then seeded 5% (vol/vol) in 10% (vol/vol) mPYG (dilute medium) with or without FAAP addition. FAAP solutions were pre-reduced ≥18 hours prior to use. Growth curves were carried out in 96-well plates (Falcon BDTM, Corning, NY, USA) in biological triplicate. Wells were overlaid with 50 µL sterile mineral oil (Sigma-Aldrich, St. Louis, MO, USA) to prevent evaporation. Plates were placed in an anaerobic chamber (Concept, Baker Ruskinn, Sanford, ME, USA; anaerobic conditions) containing an Epoch 2 microplate spectrophotometer (BioTek Instruments, Inc., Winooski, VT, USA). Plates were incubated for 48 hours at 37°C. OD_600_ readings were recorded every 30 minutes following 10 seconds of double orbital shaking.

### Growth curve analysis

Optical density measurements were blank-corrected against the first OD_600_ reading for each well. Growth curve metrics area under the curve (AUC), carrying capacity (k), maximum growth rate (r), and the mean of the three highest OD_600_ readings for each curve (ODh) were analyzed using the R package Growthcurver (53). For curves that could not be fit, k and r values were not utilized in downstream statistical analyses.

### Profiling amino acid metabolism by *Fusobacterium* strains

A total of 31 *Fusobacterium* strains to be profiled for amino acid depletion patterns were grown anaerobically at 37°C on FAA and then in pre-reduced mPYG broth (Table 2). Thereafter, *Fusobacterium* strains were seeded at 5% (vol/vol) in pre-reduced dilute medium with either casein, myosin, or soy FAAP or no FAAP treatment. *Fusobacterium* and sterile medium controls were incubated in batch culture anaerobically at 37°C. The duration of the batch culture was determined individually for each strain based on the earliest time point at which one or more FAAP treatment condition(s) reached the mean highest OD_600_ reading across three replicates. The sterile media controls were incubated for the same duration as that of the strain that last reached an initial mean highest OD_600_ reading. Thereafter, samples were collected and immediately centrifuged at 16,000 × *g* for 5 minutes at 4°C. Supernatants were filtered through sterile 0.22-µm PES syringe filters (GE Whatman, Mississauga, ON, Canada).

Filtrates were immediately prepared for 1D ^1^H nuclear magnetic resonance spectroscopy (NMR) by applying an internal standard 4,4-dimethyl-4-silapentane-1-sulfonic acid (DSS) and 0.2% (wt/vol) sodium azide in 99.9% D_2_O to a final DSS concentration of 0.5 mM. Filtered samples with DSS were transferred to 5-mm diameter glass NMR tubes, stored at 4°C, and protected from light for 24–72 hours before scanning. Samples were allowed to reach room temperature before insertion into the NMR spectrometer. Methods for spectral acquisition were as described previously (54). Briefly, samples were scanned with the first increment of ^1^H NOESY in a Bruker AVANCE 600 MHz spectrometer equipped with a 5-mm TCI Cryoprobe (NMR Centre, Advanced Analysis Centre, University of Guelph, ON, Canada). Spectra were automatically phase- and baseline-corrected in by spline fitting using TopSpin 4.0 (Buker BioSpin, Milton, ON, Canada). Sample pH was measured immediately after scanning using colorimetric indicator pH dip strips.

NMR spectra were processed and analyzed in the Chenomx software suite version 8.6. A library of 20 amino acid spectral signatures (Ala, Arg, Asn, Asp, Cys, Glu, Gln, glycine (Gly), His, Iso, Leu, Lys, Met, Phe, Pro, Ser, Thr, Trp, Tyr, and Val) was compiled from the Chenomx internal library.

### *Fusobacterium animalis* clade analysis

Ten of the 15 *F. animalis* strains tested have associated genomes (Table S5). These genomes were compared against the subset of 82 available *Fusobacterium* genomes originally used to distinguish the *F. animalis* clades, available at PRJNA937266. Genomes were input into kSNP version 4.1 (55) using a *k*-mer size of 13, and the resulting maximum-likelihood parsimony tree was visualized with iTOL version 6 (56). All 10 strains with available genomes tested were assigned to clade 2 (Fig. S5).

### Statistical analysis

To estimate growth curve characteristics, logistic regression was fitted for all 39 strains tested in all replicates of all conditions. For strains with one or more dilute medium control curve(s) that could not be fit, no comparisons between FAAP treatment and control maximum growth rate nor carrying capacity were made; if one or more FAAP treatment replicate(s) could not be fit, but all dilute medium control curves were well-fitted, the r and k values of the FAAP treatment with fewer than three well-fitted replicates were not compared to those of the control curves. Logistic regression was calculated for all growth curves and further analysis carried out only for strains with well-fitted curves, as strains without well-fitted curves lacked reliable r and k values.

Fold changes in growth curve metrics were calculated as following: (i) where the FAAP treatment value was greater than that of the no FAAP treatment condition, the FAAP treatment value was divided by the no FAAP treatment condition; or (ii) where the FAAP treatment condition value was smaller than that of the no FAAP treatment condition, the FAAP treatment value was divided by the FAAP treatment condition and multiplied by −1. Growth curve metrics between each FAAP treatment and no FAAP treatment conditions of each strain were compared by multiple *t*-tests with *P*-values false discovery rate (FDR)-corrected using the Benjamini–Hochberg (BH) method. For each growth metric of each strain with all three FAAP treatment conditions significantly differing from the no FAAP control, comparisons were also made for the given growth metric of the respective strain between FAAP treatment conditions using multiple *t*-tests with *P*-values FDR-corrected using the BH method.

Where *Fusobacterium* strains were assessed both for the effect of FAAP treatment on growth curve metrics and on amino acid metabolism by NMR spectroscopy, strain-level heterogeneity was assessed by performing separate k-means clustering (37) of the strains using their growth curve metrics, AUC and ODh, and amino acid profiles. The elbow method was used to determine the number of clusters in each case by plotting the within-cluster sum of squares against number of clusters (1–25 clusters). Strains that consistently clustered together were determined to be homogeneous in their ability to metabolize free amino acids, while the separate clustering of strains indicated cases of heterogeneity. The cluster patterns of each strain by growth curve metrics and amino acid profile were compared against the subspecies of each strain, as determined by comparing the 16S rRNA gene v3–V6 variable region sequence against the NCBI database.

## SUPPLEMENTAL MATERIAL

Supplemental material msphere.00789-24-s0001.docx Fig. S1-S5; Tables S1-S6.

ASM does not own the copyrights to Supplemental Material that may be linked to, or accessed through, an article. The authors have granted ASM a non-exclusive, world-wide license to publish the Supplemental Material files. Please contact the corresponding author directly for reuse.
