# Supplementary material for: Characterization of diet-linked amino acid pool influence on Fusobacterium spp. growth and metabolism
Source: mSphere. 2025 Feb 13;10(3):e00789-24. doi: 10.1128/msphere.00789-24 (PMC11934328; doi:10.1128/msphere.00789-24)
Supplement: Supplemental material — Fig. S1-S5; Tables S1-S6. [file msphere.00789-24-s0001.docx]

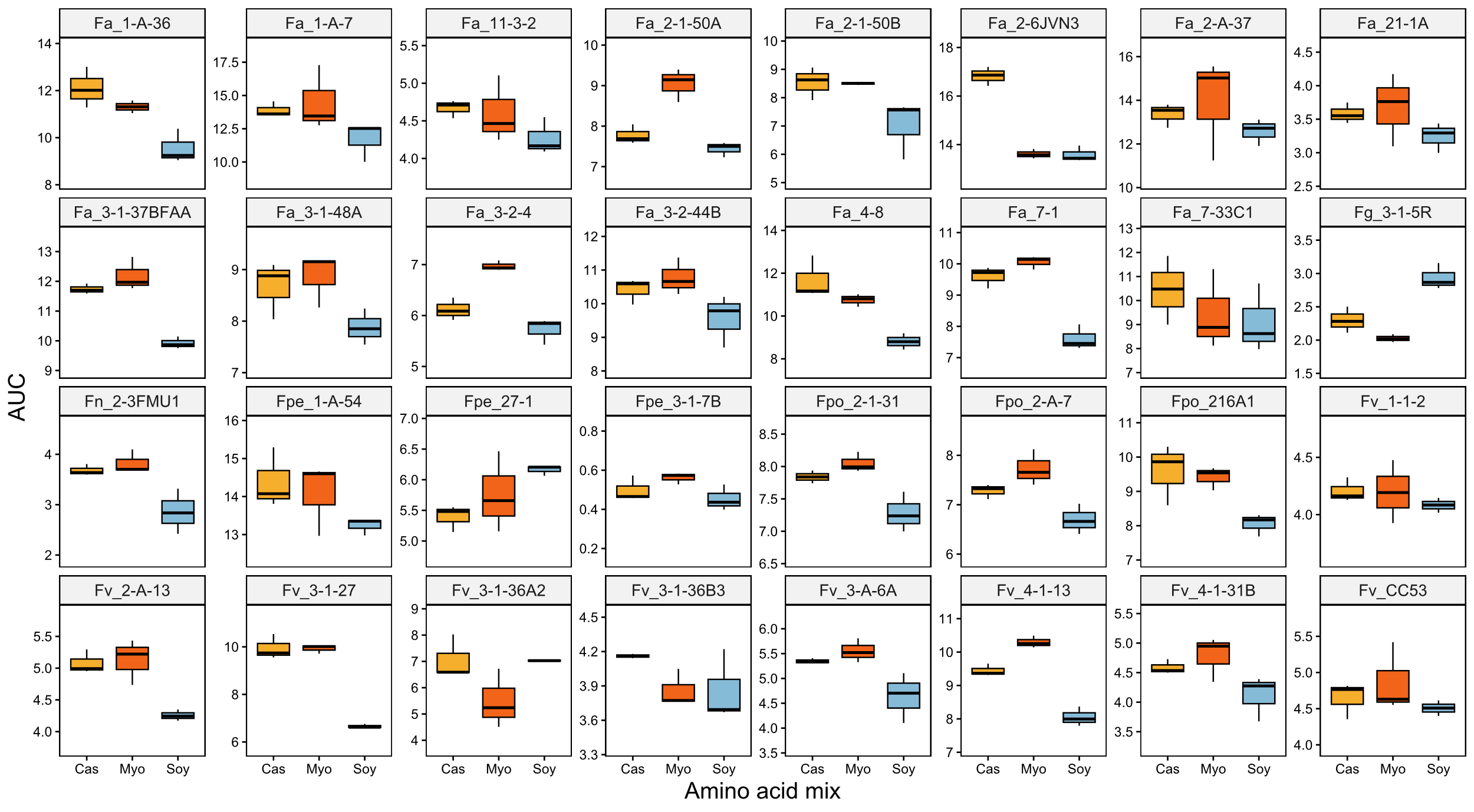

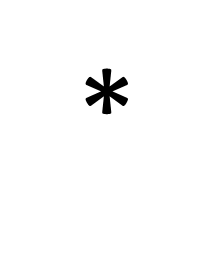

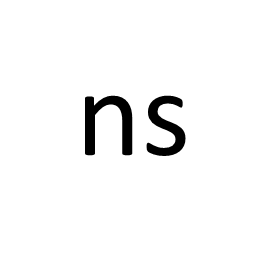

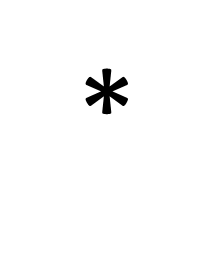

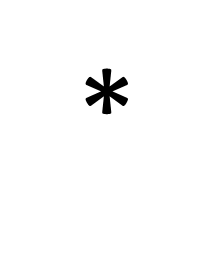

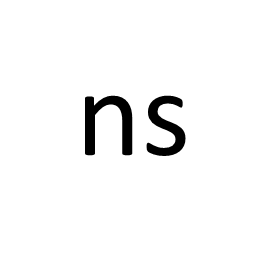

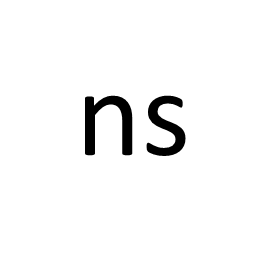

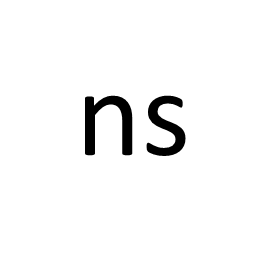

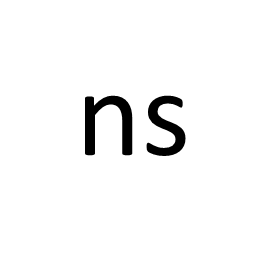

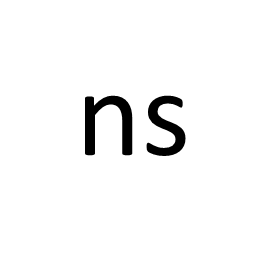

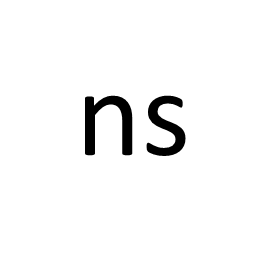

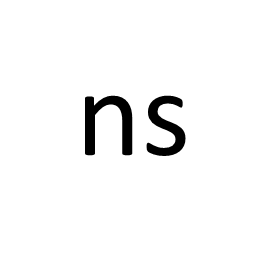

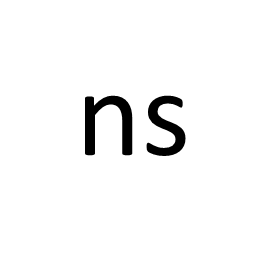

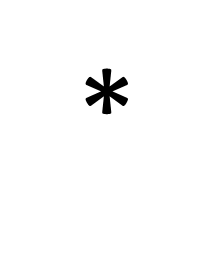

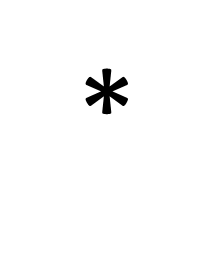

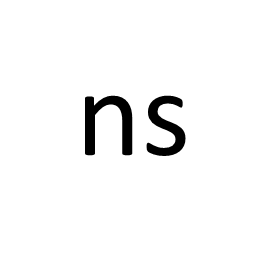

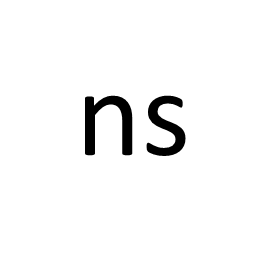

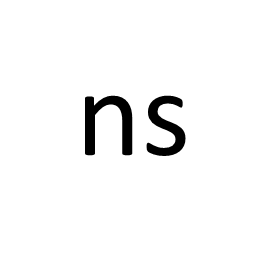

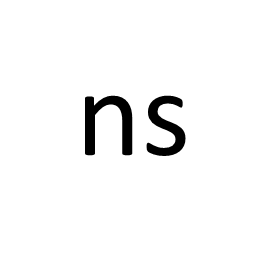

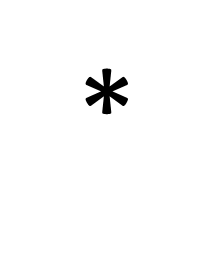

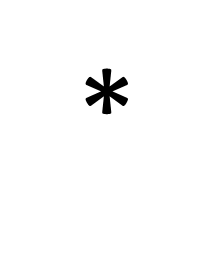

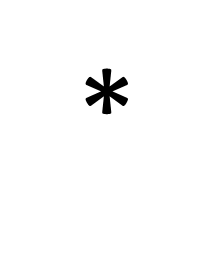

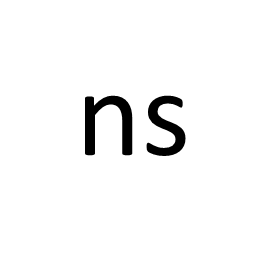

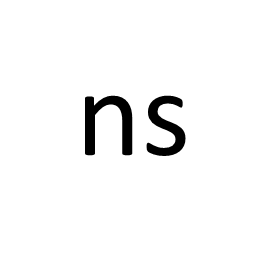

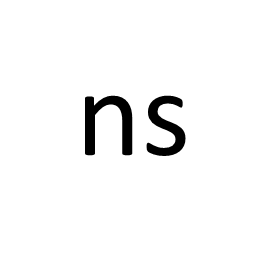

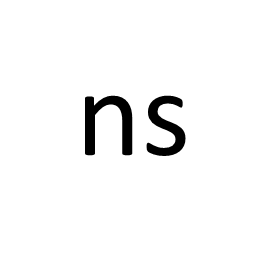

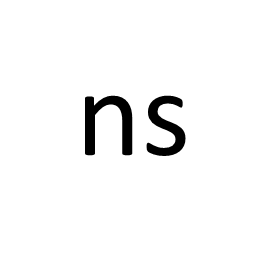

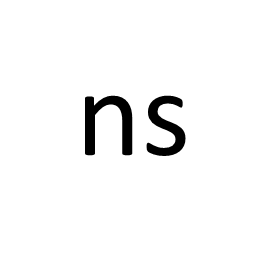

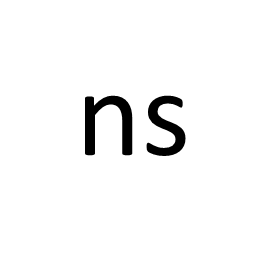

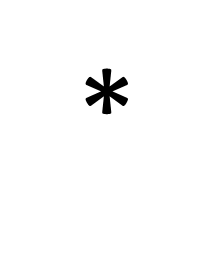

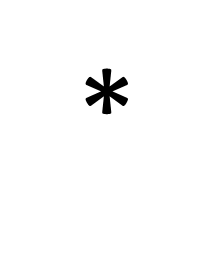

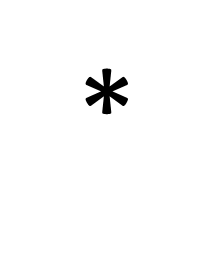

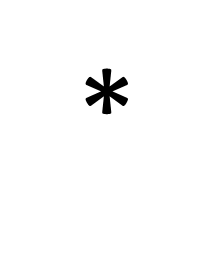

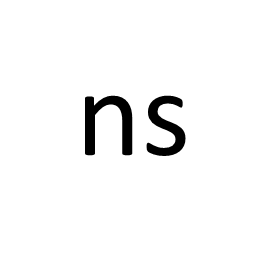

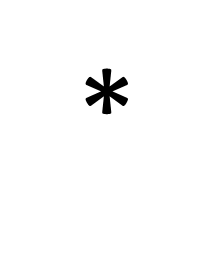

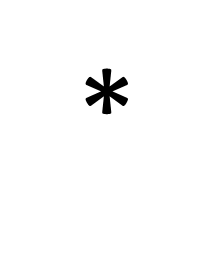

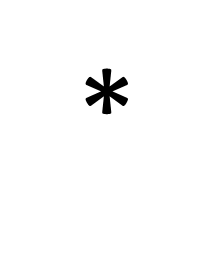

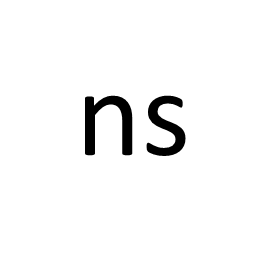

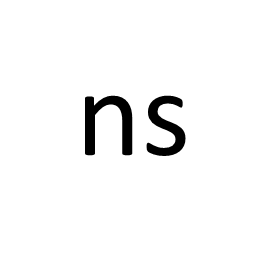

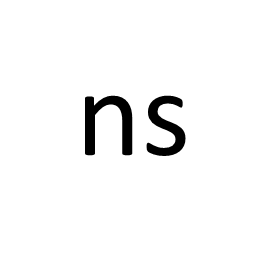

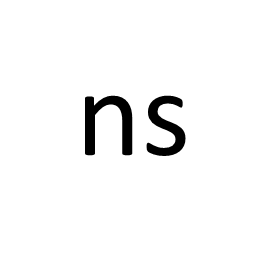

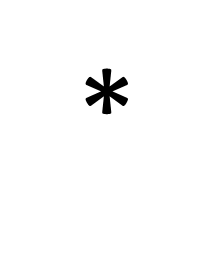

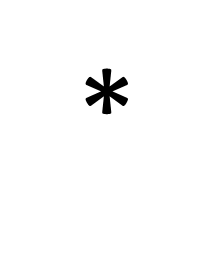

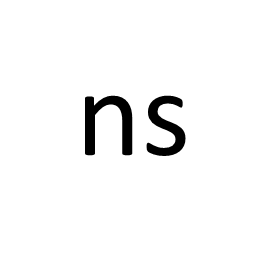

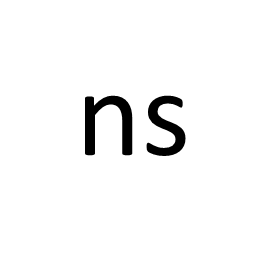

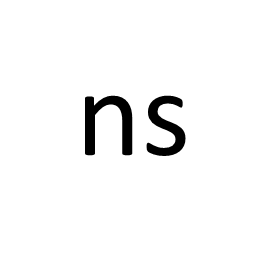

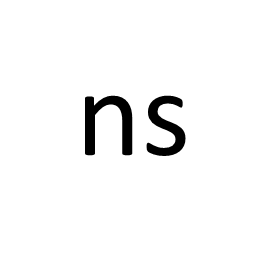

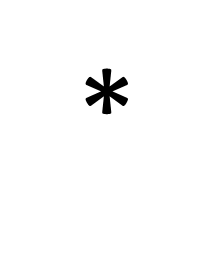

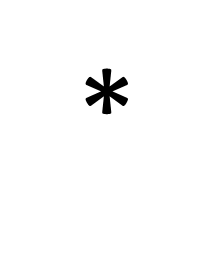

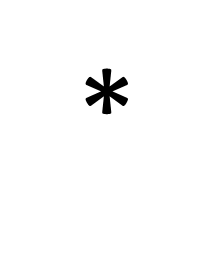

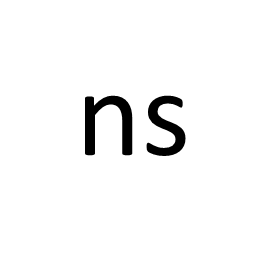

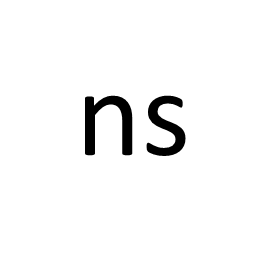

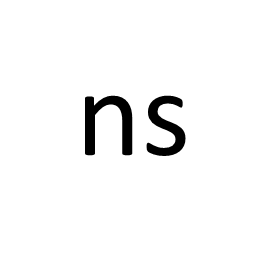

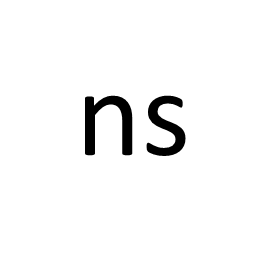

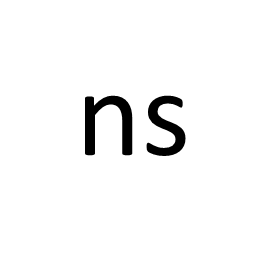

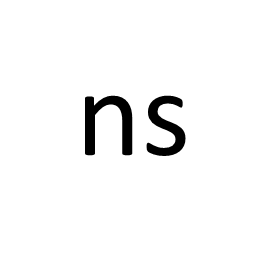

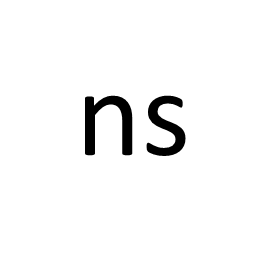

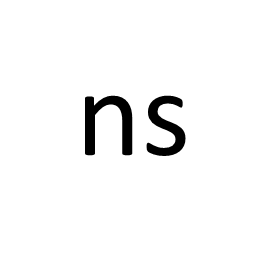

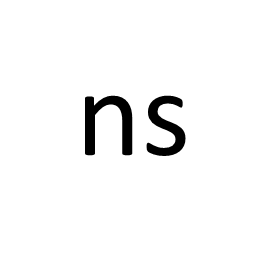

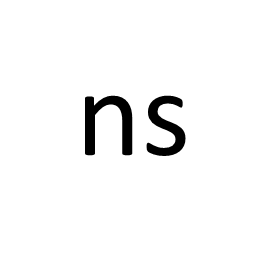

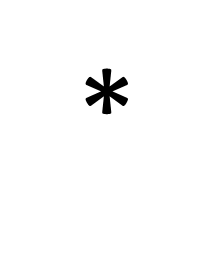

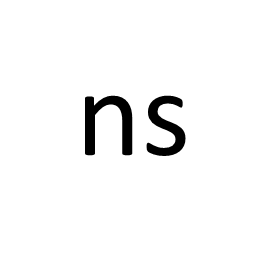

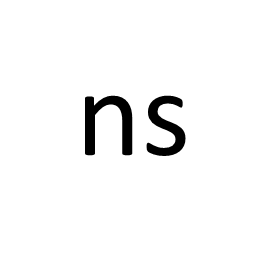

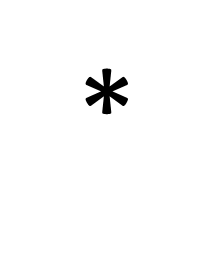

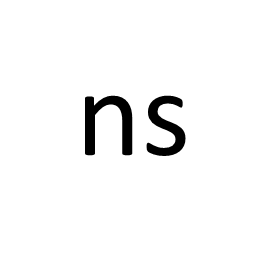

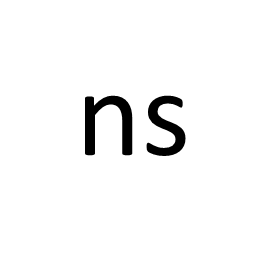

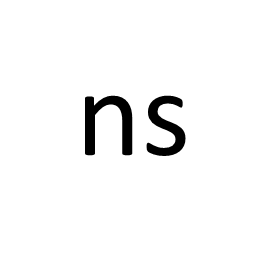

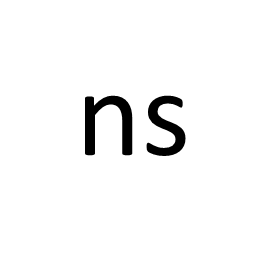

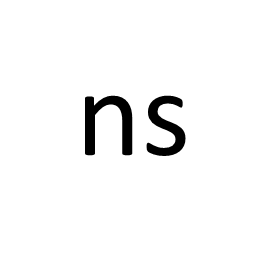

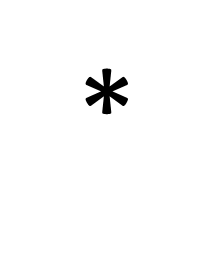

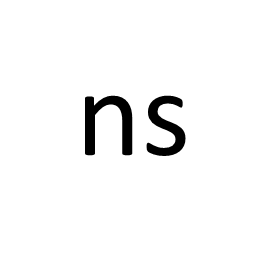

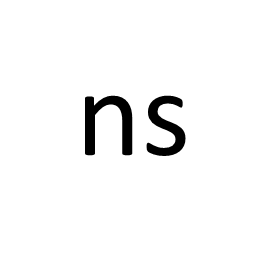

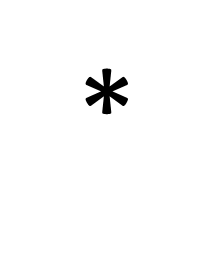

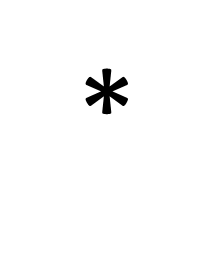

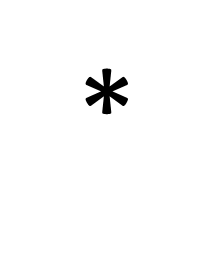

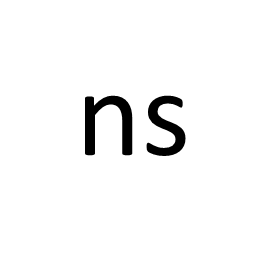

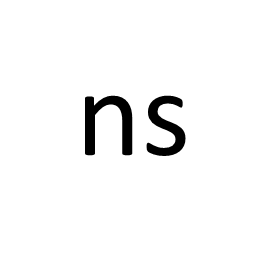

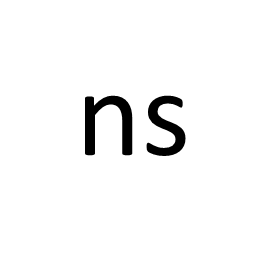

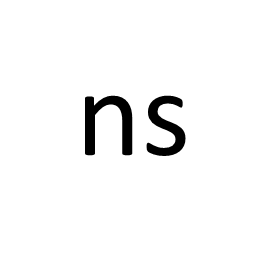

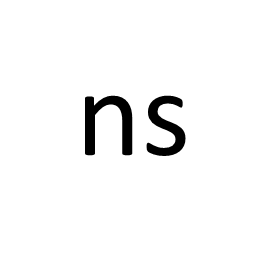

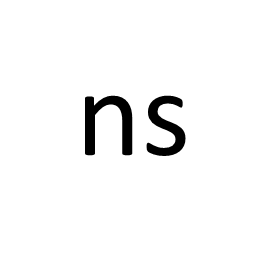

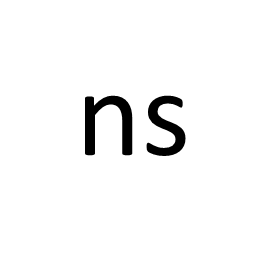

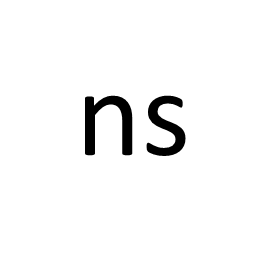

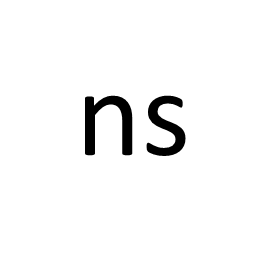

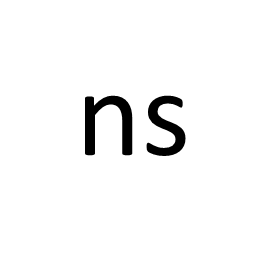

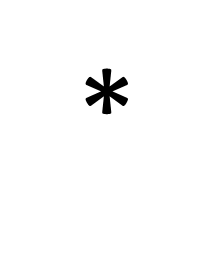

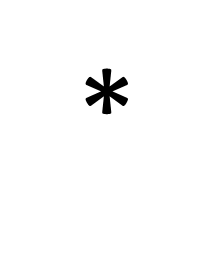

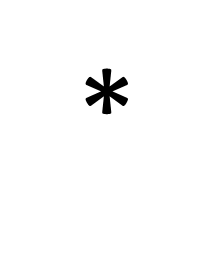

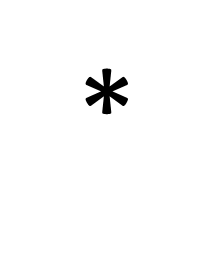

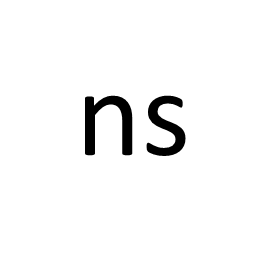

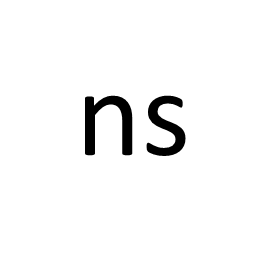

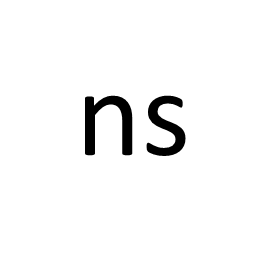

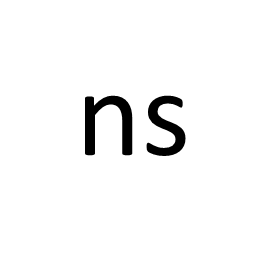

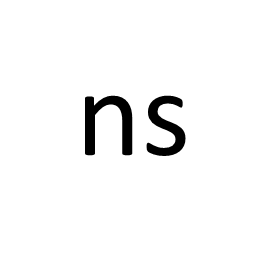

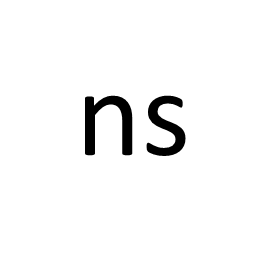


**Figure S1:** Boxplots of areas under the curve (AUC) produced following treatment with casein (cas), myosin (myo), or soy free amino acid pools (FAAPs) for each strain of *Fusobacterium* spp. where all FAAP treatments were significantly different from controls. Abbreviations: Fa: *F. animalis*; Fg: *F. gonidiaformans*; Fn: *F. nucleatum*; Fpe: *F. periodonticum*; Fpo: *F. polymorphum*; Fv: *F. vincentii*. T-tests with Benjamini-Hochberg false discovery rate corrections were conducted in R using the package rstatix. Corrected p-values are shown by asterisks as follows: **** = p-value < 0.0001; *** = p-value < 0.001; ** = p-value < 0.01; * = p-value < 0.05; ns = not significant.


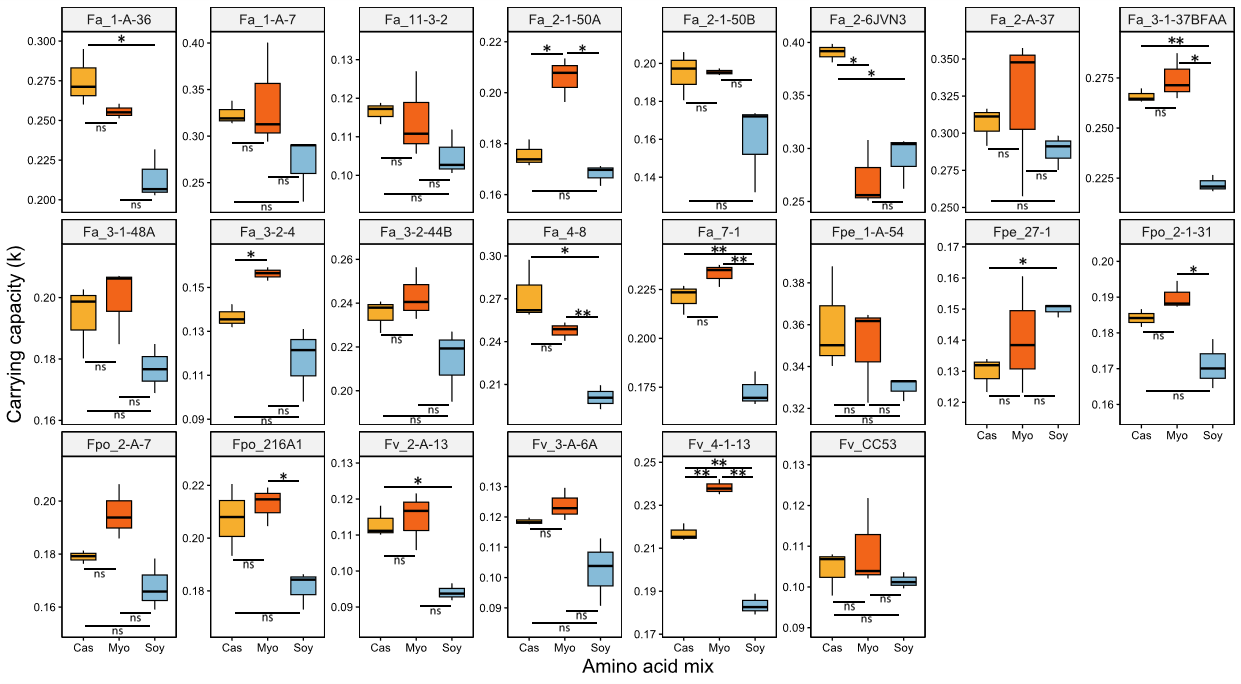


**Figure S2:** Boxplots of carrying capacities (k) produced following treatment with casein (cas), myosin (myo), or soy free amino acid pools (FAAPs) for each strain of *Fusobacterium* spp. where all FAAP treatments were significantly different from controls. Abbreviations: Fa: *F. animalis*; Fn: *F. nucleatum*; Fpe: *F. periodonticum*; Fpo: *F. polymorphum*; Fv: *F. vincentii*. T-tests with Benjamini-Hochberg false discovery rate corrections were conducted in R using the package rstatix. Corrected p-values are shown by asterisks as follows: **** = p-value < 0.0001; *** = p-value < 0.001; ** = p-value < 0.01; * = p-value < 0.05; ns = not significant.


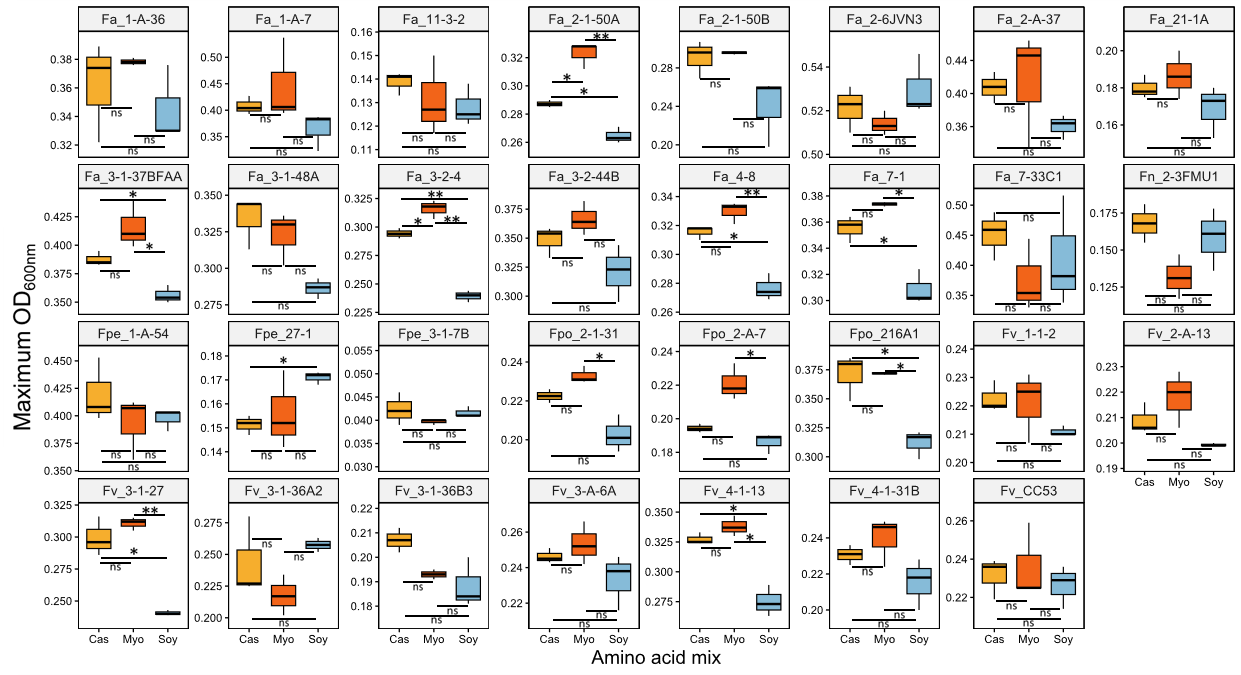


**Figure S3:** Boxplots of the three maximum optical density (OD_600nm_) readings produced following treatment with casein (cas), myosin (myo), or soy free amino acid pools (FAAPs) for each strain of *Fusobacterium* spp. where all FAAP treatments were significantly different from controls. Abbreviations: Fa: *F. animalis*; Fn: *F. nucleatum*; Fpe: *F. periodonticum*; Fpo: *F. polymorphum*; Fv: *F. vincentii*. T-tests with Benjamini-Hochberg false discovery rate corrections were conducted in R using the package rstatix. Corrected p-values are shown by asterisks as follows: **** = p-value < 0.0001; *** = p-value < 0.001; ** = p-value <0 .01; * = p-value < 0.05; ns = not significant.


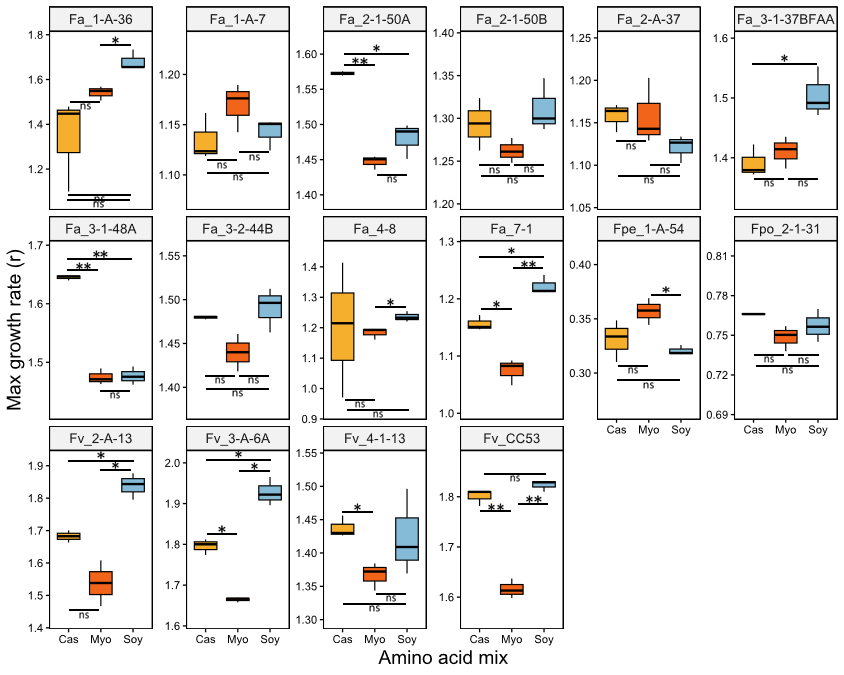


**Figure S4:** Boxplots of maximum growth rates (r) produced following treatment with casein (cas), myosin (myo), or soy free amino acid pools (FAAPs) for each strain of *Fusobacterium* spp. where all FAAP treatments were significantly different from controls. Abbreviations: Fa: *F. animalis*; Fn: *F. nucleatum*; Fpe: *F. periodonticum*; Fpo: *F. polymorphum*; Fv: *F. vincentii*. T-tests with Benjamini-Hochberg false discovery rate corrections were conducted in R using the package rstatix. Corrected p-values are shown by asterisks as follows: **** = p-value < 0.0001; *** = p-value < 0.001; ** = p-value < 0.01; * = p-value < 0.05; ns = not significant.


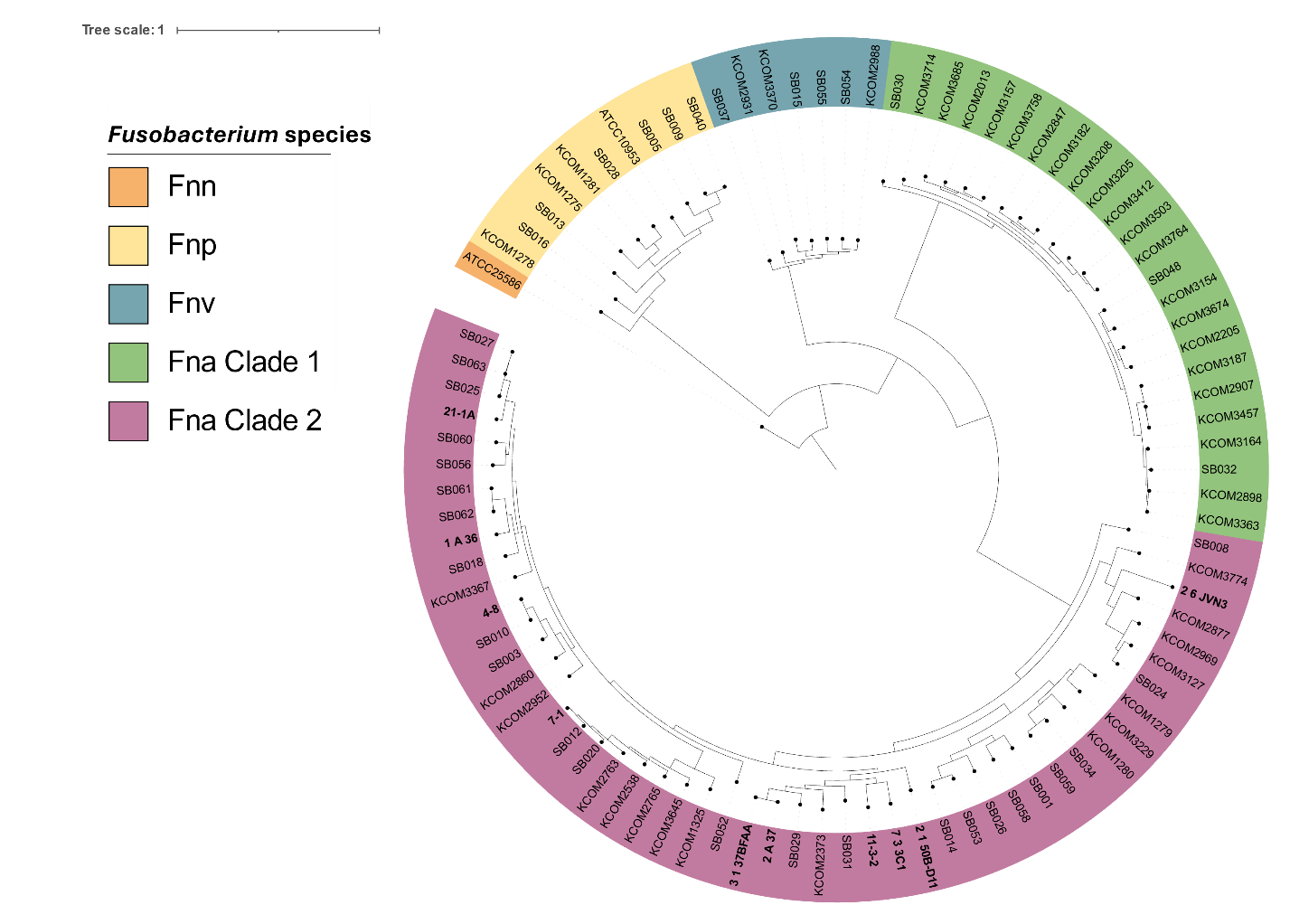


**Figure S5:** Maximum-likelihood parsimony tree of *Fusobacterium* species. Tree was created with kSNP and visualized with iTOL. Bolded strain names represent genomes of *F. animalis* strains from this paper.

**Table S1:** *Fusobacterium* spp. strains and free amino acid pool (FAAP) treatments from which single biological replicate outliers were removed from downstream analyses due to high aggregation.

| Species | Strain | FAAP treatment |
| --- | --- | --- |
| *F. necrophorum* | 1-1-36S | Casein |
|  |  | Soy |
|  | 3-1-49 | Casein |
|  |  | Myosin |
| *F. polymorphum* | 2-1-31 | Casein |
| *F. vincentii* | 3-1-36A2 | Soy |
|  | 3-1-36B3 | Casein |

**Table S2:** The timepoint in hours at which each strain of *Fusobacterium* spp. reached an initial maximum optical density (OD_600nm_) reading in one of the three free amino acid pool treatments. This first timepoint at which the maximum OD_600nm_ was reached was selected for analysis of initial free amino acid depletion by each *Fusobacterium* spp. strain.

| ***Fusobacterium* spp.** | **Strain** | **Max OD_600nm_ timepoint (hr)** |
| --- | --- | --- |
| *F. animalis* | 3-2-4 | 8 |
|  | 3-2-44B | 8 |
|  | 1-A-36 | 8.5 |
|  | 3-1-37BFAA | 8.5 |
|  | 3-1-48A | 8.5 |
|  | 7_33C1 | 9 |
|  | 2-1-50A | 9 |
|  | 4-8 | 9 |
|  | 2-1-50B | 9.5 |
|  | 21-1A | 9.5 |
|  | 2-A-37 | 9.5 |
|  | 1-A-7 | 10 |
|  | 2_6JVN3 | 10 |
|  | 7-1 | 10.5 |
|  | 11-3-2 | 22 |
| *F. nucleatum* | 2_3FMU1 | 15 |
| *F. polymorphum* | 4-A-7 | 5 |
|  | 216A1 | 9 |
|  | 1-A-13 | 11.5 |
|  | 2-A-7 | 13.5 |
|  | 2-1-31 | 16.5 |
|  | 13-3C | 48 |
| *F. vincentii* | 4-1-13 | 9 |
|  | 3-A-6A | 7.5 |
|  | 2-A-13 | 8 |
|  | 4-1-31B | 8.5 |
|  | CC53 | 8.5 |
|  | 3-1-27 | 9 |
|  | 1-1-2 | 9.5 |
|  | 3-1-36A2 | 9.5 |
|  | 3-1-36B3 | 11 |

**Table S3:** The pH value of *Fusobacterium* spp. culture supernatants following culture in control medium, and control medium with free amino acid pool (FAAP) treatments mimicking casein, myosin, and soy proteins. The pH values of culture supernatants were determined immediately after supernatants were subjected to one-dimensional proton nuclear magnetic spectroscopy (1D ^1^H NMR) for detection of amino acid depletion.

| *Fusobacterium* spp. | Strain | Control pH | Casein FAAP pH | Myosin FAAP pH | Soy FAAP pH |
| --- | --- | --- | --- | --- | --- |
| *F. animalis* | 2-1-50A | 6.5 | 7.5 | 7.5 | 7.5 |
|  | 3-2-4 | 6.5 | 7.5 | 7.5 | 7.5 |
|  | 1-A-7 | 6.5 | 7.5 | 7.5 | 7.5 |
|  | 1-A-36 | 6.5 | 7.5 | 7.5 | 7.5 |
|  | 2-A-37 | 6.5 | 7.5 | 7.5 | 7.5 |
|  | 3-1-37BFAA | 6.5 | 7.5 | 7.5 | 7.5 |
|  | 3-2-44B | 6.5 | 7.5 | 7.5 | 7.5 |
|  | 3-1-48A | 6.5 | 7.5 | 7.5 | 7.5 |
|  | 2-1-50B | 6.5 | 7.5 | 7.5 | 7.5 |
|  | 2-6JVN3 | 6.5 | 7.5 | 7.5 | 7.5 |
|  | 4-8 | 6.5 | 7.5 | 7.5 | 7.5 |
|  | 7-1 | 6.5 | 7.5 | 7.5 | 7.5 |
|  | 11-3-2 | 6.5 | 7.5 | 7.5 | 7.5 |
|  | 21-1A | 6.5 | 7.5 | 7.5 | 7.5 |
|  | 7-33C1 | 6.5 | 7.5 | 7.5 | 7.5 |
| *F. nucleatum* | 2-3FMU1 | 6.5 | 7.5 | 7.5 | 7.5 |
| *F. polymorphum* | 13-3C | 6.5 | 7.5 | 7.5 | 7.5 |
|  | 2-A-7 | 6.5 | 7.5 | 7.5 | 7.5 |
|  | 4-A-7 | 6.5 | 7.5 | 7.5 | 7.5 |
|  | 1-A-13 | 6.5 | 7.5 | 7.5 | 7.5 |
|  | 2-1-31 | 6.5 | 7.5 | 7.5 | 7.5 |
|  | 216A1 | 6.5 | 7.5 | 7.5 | 7.5 |
| *F. vincentii* | 4-1-13 | 6.5 | 7.5 | 7.5 | 7.5 |
|  | 2-A-13 | 6.5 | 7.5 | 7.5 | 7.5 |
|  | 3-1-27 | 6.5 | 7.5 | 7.5 | 7.5 |
|  | 4-1-31B | 6.5 | 7.5 | 7.5 | 7.5 |
|  | 3-1-36B3 | 6.5 | 7.5 | 7.5 | 7.5 |
|  | 3-1-36A2 | 6.5 | 7.5 | 7.5 | 7.5 |
|  | 1-1-2 | 6.5 | 7.5 | 7.5 | 7.5 |
|  | 3-A-6A | 6.5 | 7.5 | 7.5 | 7.5 |
|  | CC53 | 6.5 | 7.5 | 7.5 | 7.5 |
| Sterile controls | - | 7.0 | 7.0 | 7.0 | 7.0 |

**Table S4:** Utilization of each amino acid in the free amino acid pool (FAAP) treatments by each strain of *Fusobacterium nucleatum* as measured by 1D proton NMR spectroscopy. Amino acid utilization was calculated from concentrations of each amino acid detected in the supernatant following batch culture of each strain in each FAAP treatment compared to the amino acid concentrations detected in the sterile FAAP-supplemented medium. Amino acid utilizations by each *F. nucleatum* strain are expressed as decimals. Abbreviations: A: alanine; R: arginine; N: asparagine; D: aspartate; E: glutamate; Q: glutamine; G: glycine; I: isoleucine; L: leucine; K: lysine; M: methionine; F: phenylalanine; P: proline; S: serine; T: threonine; W: tryptophan; Y: tyrosine; V: valine.

| FAAP treatment | *Fusobacterium* spp. | Strain | A | R | N | D | E | Q | G | I | L | K | M | F | P | S | T | W | Y | V |
| --- | --- | --- | --- | --- | --- | --- | --- | --- | --- | --- | --- | --- | --- | --- | --- | --- | --- | --- | --- | --- |
| Casein | *F. animalis* | 1-A-7 | -0.247 | 0.375 | 0.281 | 0.292 | 0.700 | 0.228 | -0.145 | 0.005 | 0.007 | 0.671 | 0.321 | 0.004 | 0.113 | 0.737 | 0.488 | 0.364 | 0.141 | 0.051 |
|  |  | 1-A-36 | -0.391 | 0.281 | 0.191 | 0.247 | 0.826 | 0.048 | -0.302 | 0.044 | 0.024 | 0.664 | 0.182 | 0.034 | 0.138 | 0.637 | 0.338 | 0.324 | 0.014 | -0.086 |
|  |  | 2_6JVN3 | -0.500 | 0.727 | 0.292 | 0.230 | 0.937 | 0.677 | -0.306 | -0.030 | 0.063 | 0.951 | 0.306 | 0.075 | 0.067 | 0.722 | 0.374 | 0.139 | 0.030 | -0.043 |
|  |  | 2-A-37 | -0.284 | 0.269 | 0.346 | 0.411 | 0.911 | 0.418 | -0.165 | 0.078 | 0.129 | 0.880 | 0.616 | 0.048 | 0.083 | 0.918 | 0.659 | 0.531 | 0.090 | 0.084 |
|  |  | 7_33C1 | -0.171 | 0.284 | 0.286 | 0.277 | 0.714 | -0.048 | -0.116 | 0.176 | 0.143 | 0.601 | 0.367 | 0.069 | 0.151 | 0.642 | 0.459 | 0.425 | 0.098 | 0.078 |
|  |  | 3-1-37BFAA | -0.317 | 0.214 | 0.202 | 0.218 | 0.664 | -0.028 | -0.202 | 0.048 | 0.071 | 0.616 | 0.277 | 0.006 | 0.090 | 0.578 | 0.298 | 0.332 | 0.072 | -0.019 |
|  |  | 3-2-44B | -0.251 | 0.135 | 0.233 | 0.226 | 0.672 | -0.174 | -0.177 | 0.214 | 0.138 | 0.611 | 0.315 | 0.027 | 0.102 | 0.559 | 0.332 | 0.385 | 0.038 | -0.019 |
|  |  | 3-1-48A | -0.269 | 0.274 | 0.145 | 0.175 | 0.653 | -0.252 | -0.237 | 0.134 | 0.053 | 0.575 | 0.230 | 0.041 | 0.105 | 0.535 | 0.236 | 0.390 | 0.017 | -0.062 |
|  |  | 2-1-50A | -0.164 | 0.544 | 0.339 | 0.201 | 0.792 | 0.101 | -0.140 | 0.189 | 0.106 | 0.684 | 0.372 | 0.196 | 0.220 | 0.747 | 0.520 | 0.403 | 0.060 | 0.066 |
|  |  | 2-1-50B | -0.384 | 0.258 | 0.108 | 0.204 | 0.917 | 0.331 | -0.400 | 0.102 | -0.018 | 0.803 | 0.421 | 0.135 | 0.110 | 0.971 | 0.555 | 0.383 | 0.045 | -0.101 |
|  |  | 11-3-2 | -0.184 | 0.211 | 0.209 | 0.126 | 0.906 | 0.049 | -0.104 | 0.017 | 0.039 | 0.569 | 0.245 | -0.022 | 0.040 | 0.947 | 0.674 | 0.393 | 0.024 | 0.065 |
|  |  | 3-2-4 | -0.196 | 0.284 | 0.059 | 0.181 | 0.901 | -0.268 | -0.160 | 0.114 | 0.043 | 0.826 | 0.249 | 0.018 | 0.078 | 0.707 | 0.175 | 0.237 | 0.058 | -0.012 |
|  |  | 21-1A | -0.215 | 0.308 | 0.217 | 0.215 | 0.732 | 0.358 | -0.174 | 0.037 | 0.059 | 0.670 | 0.213 | 0.057 | 0.128 | 0.838 | 0.437 | 0.336 | 0.067 | 0.027 |
|  |  | 4-8 | -0.359 | 0.222 | 0.317 | 0.421 | 0.925 | 0.767 | -0.277 | 0.109 | 0.067 | 0.992 | 0.656 | 0.120 | 0.113 | 0.986 | 0.980 | 0.555 | 0.104 | 0.008 |
|  |  | 7-1 | -0.373 | 0.160 | 0.329 | 0.210 | 0.812 | 0.039 | -0.239 | 0.165 | 0.037 | 0.776 | 0.476 | -0.007 | 0.061 | 0.832 | 0.543 | 0.496 | 0.025 | 0.007 |
|  | *F. nucleatum* | 2_3FMU1 | -0.189 | 0.248 | 0.058 | 0.057 | 0.229 | -0.051 | -0.229 | 0.211 | 0.009 | 0.478 | 0.122 | 0.058 | 0.056 | 0.714 | 0.260 | 0.322 | 0.016 | -0.036 |
|  | *F. polymorphum* | 1-A-13 | -0.211 | 0.223 | 0.114 | -0.006 | 0.104 | 0.172 | -0.224 | 0.064 | 0.009 | 0.318 | 0.100 | 0.090 | 0.056 | 0.292 | -0.077 | 0.360 | -0.001 | -0.063 |
|  |  | 2-A-7 | -0.185 | 0.240 | 0.127 | 0.025 | 0.623 | -0.196 | -0.163 | 0.067 | 0.044 | 0.392 | 0.129 | 0.074 | 0.092 | 0.326 | -0.013 | 0.354 | 0.079 | 0.006 |
|  |  | 4-A-7 | 0.133 | 0.320 | 0.264 | 0.300 | 0.194 | -0.287 | 0.115 | 0.215 | 0.175 | 0.373 | 0.322 | 0.183 | 0.250 | 0.339 | 0.190 | 0.332 | 0.233 | 0.227 |
|  |  | 216A1 | -0.230 | 0.254 | 0.297 | 0.302 | 0.767 | 0.121 | -0.120 | 0.198 | 0.063 | 0.779 | 0.649 | 0.063 | 0.095 | 0.797 | 0.524 | 0.504 | 0.050 | 0.064 |
|  |  | 2-1-31 | -0.510 | 0.410 | 0.616 | -0.010 | 0.700 | 1.000 | -0.411 | 0.105 | 0.039 | 0.986 | 0.291 | 0.110 | 0.159 | 0.571 | 0.027 | 0.587 | 0.094 | -0.027 |
|  |  | 13-3C | -0.084 | 0.225 | 0.194 | 0.095 | 0.163 | -0.338 | -0.075 | 0.027 | 0.038 | 0.194 | 0.161 | 0.071 | 0.090 | 0.450 | 0.238 | 0.250 | 0.059 | 0.083 |
|  | *F. vincentii* | 2-A-13 | -0.286 | 0.199 | 0.227 | 0.156 | 0.724 | 0.264 | -0.229 | 0.003 | 0.100 | 0.629 | 0.222 | -0.013 | 0.121 | 0.743 | 0.433 | 0.158 | 0.002 | -0.035 |
|  |  | 3-A-6A | -0.141 | 0.285 | 0.235 | 0.169 | 0.641 | 0.266 | -0.098 | 0.056 | 0.063 | 0.599 | 0.187 | 0.011 | 0.118 | 0.711 | 0.386 | 0.285 | 0.071 | 0.064 |
|  |  | CC53 | -0.180 | 0.269 | 0.210 | 0.179 | 0.452 | 0.121 | -0.127 | 0.124 | 0.036 | 0.446 | 0.108 | 0.020 | 0.121 | 0.551 | 0.379 | 0.394 | 0.027 | 0.017 |
|  |  | 4-1-31B | -0.191 | 0.163 | 0.026 | 0.153 | 0.445 | -0.094 | -0.242 | 0.081 | 0.059 | 0.437 | 0.222 | 0.035 | 0.095 | 0.566 | 0.175 | 0.308 | 0.088 | -0.030 |
|  |  | 3-1-36A2 | -0.106 | 0.249 | 0.192 | 0.138 | 0.508 | 0.157 | -0.061 | 0.062 | 0.069 | 0.374 | 0.162 | 0.042 | 0.133 | 0.730 | 0.430 | 0.274 | 0.035 | 0.073 |
|  |  | 3-1-36B3 | -0.234 | 0.167 | 0.258 | 0.160 | 0.833 | 0.633 | -0.200 | 0.017 | 0.047 | 0.684 | 0.115 | 0.008 | 0.072 | 0.963 | 0.745 | 0.333 | 0.048 | 0.019 |
|  |  | 1-1-2 | -0.128 | 0.106 | 0.237 | 0.135 | 0.616 | -0.059 | -0.074 | 0.067 | 0.012 | 0.333 | 0.177 | 0.038 | 0.093 | 0.833 | 0.435 | 0.283 | 0.087 | 0.078 |
|  |  | 4-1-13 | -0.163 | 0.243 | 0.305 | 0.257 | 0.596 | -0.189 | -0.083 | 0.070 | 0.066 | 0.604 | 0.418 | 0.058 | 0.174 | 0.575 | 0.495 | 0.450 | 0.063 | 0.082 |
|  |  | 3-1-27 | -0.141 | 0.270 | 0.170 | 0.160 | 0.495 | 0.059 | -0.110 | 0.101 | 0.066 | 0.397 | 0.177 | 0.036 | 0.137 | 0.584 | 0.347 | 0.297 | 0.069 | 0.053 |
| Myosin | *F. animalis* | 1-A-7 | -0.118 | 0.435 | 0.260 | 0.238 | 0.709 | 0.409 | -0.056 | 0.093 | 0.060 | 0.597 | 0.296 | 0.018 | 0.036 | 0.840 | 0.555 | 0.229 | 0.068 | 0.017 |
|  |  | 1-A-36 | -0.258 | 0.222 | 0.107 | 0.172 | 0.744 | 0.293 | -0.214 | 0.086 | 0.034 | 0.542 | 0.176 | 0.082 | -0.004 | 0.722 | 0.416 | 0.228 | 0.005 | -0.117 |
|  |  | 2_6JVN3 | -0.344 | 0.853 | 0.364 | 0.178 | 0.897 | 0.729 | -0.166 | 0.090 | 0.120 | 0.797 | 0.365 | 0.025 | -0.011 | 0.806 | 0.522 | 0.269 | 0.126 | -0.080 |
|  |  | 2-A-37 | -0.213 | 0.161 | 0.212 | 0.287 | 0.971 | 0.721 | -0.114 | 0.185 | 0.047 | 0.719 | 0.574 | 0.099 | -0.029 | 0.908 | 0.675 | 0.363 | 0.170 | -0.029 |
|  |  | 7_33C1 | -0.120 | 0.149 | 0.201 | 0.200 | 0.643 | 0.317 | -0.074 | 0.173 | 0.051 | 0.528 | 0.324 | 0.077 | 0.000 | 0.712 | 0.500 | 0.292 | 0.155 | -0.018 |
|  |  | 3-1-37BFAA | -0.098 | 0.147 | 0.349 | 0.188 | 0.616 | 0.437 | -0.014 | 0.132 | 0.035 | 0.507 | 0.276 | 0.078 | 0.034 | 0.667 | 0.528 | 0.151 | 0.153 | 0.060 |
|  |  | 3-2-44B | -0.108 | 0.145 | 0.153 | 0.163 | 0.638 | 0.239 | -0.054 | 0.033 | 0.022 | 0.466 | 0.270 | 0.019 | 0.057 | 0.588 | 0.434 | 0.165 | 0.086 | -0.011 |
|  |  | 3-1-48A | -0.097 | 0.130 | 0.139 | 0.148 | 0.639 | 0.278 | -0.050 | 0.070 | 0.019 | 0.546 | 0.226 | 0.041 | 0.035 | 0.718 | 0.391 | 0.169 | 0.119 | -0.014 |
|  |  | 2-1-50A | -0.093 | 0.257 | 0.242 | 0.139 | 0.718 | 0.475 | -0.060 | 0.120 | 0.067 | 0.591 | 0.282 | 0.123 | 0.013 | 0.786 | 0.559 | 0.248 | 0.095 | -0.015 |
|  |  | 2-1-50B | -0.104 | 0.279 | 0.230 | 0.142 | 0.898 | 0.541 | -0.105 | 0.050 | 0.110 | 0.483 | 0.377 | 0.161 | 0.046 | 0.958 | 0.643 | 0.324 | 0.160 | -0.004 |
|  |  | 11-3-2 | -0.189 | 0.162 | 0.302 | 0.052 | 0.828 | 0.448 | -0.172 | 0.098 | 0.060 | 0.405 | 0.230 | 0.049 | -0.037 | 0.946 | 0.761 | 0.153 | 0.113 | -0.074 |
|  |  | 3-2-4 | -0.084 | 0.121 | 0.082 | 0.116 | 0.840 | 0.320 | -0.069 | 0.102 | 0.029 | 0.600 | 0.248 | 0.012 | 0.028 | 0.754 | 0.321 | 0.179 | 0.141 | -0.025 |
|  |  | 21-1A | -0.082 | 0.184 | 0.152 | 0.159 | 0.694 | 0.590 | -0.108 | 0.126 | 0.027 | 0.510 | 0.240 | 0.087 | 0.061 | 0.807 | 0.488 | 0.033 | 0.176 | -0.027 |
|  |  | 4-8 | -0.183 | 0.107 | 0.180 | 0.232 | 0.976 | 0.728 | -0.139 | 0.093 | 0.046 | 0.752 | 0.443 | 0.047 | 0.028 | 0.980 | 0.803 | 0.469 | 0.162 | -0.051 |
|  |  | 7-1 | -0.212 | 0.037 | 0.303 | 0.153 | 0.724 | 0.506 | -0.092 | 0.032 | 0.025 | 0.548 | 0.501 | -0.017 | -0.024 | 0.815 | 0.670 | 0.294 | 0.103 | -0.012 |
|  | *F. nucleatum* | 2_3FMU1 | -0.219 | 0.102 | -0.141 | 0.006 | 0.185 | 0.239 | -0.225 | 0.027 | 0.016 | 0.198 | 0.064 | 0.073 | -0.024 | 0.609 | 0.120 | 0.105 | 0.014 | -0.220 |
|  | *F. polymorphum* | 1-A-13 | -0.073 | 0.034 | 0.161 | 0.017 | 0.000 | 0.699 | -0.092 | 0.016 | 0.047 | 0.177 | 0.138 | 0.113 | -0.018 | 0.393 | 0.163 | 0.065 | 0.099 | -0.016 |
|  |  | 2-A-7 | -0.063 | 0.126 | 0.142 | 0.058 | 0.148 | 0.084 | -0.056 | 0.027 | 0.070 | 0.072 | 0.064 | -0.018 | -0.026 | 0.274 | 0.133 | 0.059 | 0.084 | -0.015 |
|  |  | 4-A-7 | -0.031 | 0.135 | 0.147 | 0.076 | 0.143 | 0.020 | -0.043 | 0.010 | 0.030 | 0.102 | 0.079 | 0.018 | -0.027 | 0.158 | 0.124 | 0.069 | 0.071 | 0.003 |
|  |  | 216A1 | -0.208 | 0.134 | 0.299 | 0.219 | 0.813 | 0.496 | -0.127 | 0.156 | 0.054 | 0.643 | 0.696 | 0.066 | 0.102 | 0.843 | 0.635 | 0.349 | 0.157 | -0.058 |
|  |  | 2-1-31 | -0.237 | 0.433 | 0.534 | -0.003 | 0.653 | 1.000 | -0.214 | 0.099 | 0.010 | 0.990 | 0.338 | 0.061 | -0.234 | 0.704 | 0.196 | 0.707 | 0.098 | -0.046 |
|  |  | 13-3C | -0.056 | 0.090 | 0.093 | 0.059 | 0.158 | 0.018 | -0.054 | 0.000 | 0.002 | 0.100 | 0.084 | -0.007 | -0.010 | 0.415 | 0.205 | -0.094 | 0.096 | -0.016 |
|  | *F. vincentii* | 2-A-13 | -0.112 | 0.120 | 0.231 | 0.123 | 0.596 | 0.642 | -0.070 | 0.039 | 0.073 | 0.473 | 0.208 | 0.021 | -0.019 | 0.821 | 0.575 | 0.040 | 0.106 | -0.012 |
|  |  | 3-A-6A | -0.097 | 0.070 | 0.093 | 0.055 | 0.469 | 0.649 | -0.094 | -0.027 | 0.049 | 0.363 | 0.138 | -0.068 | -0.039 | 0.696 | 0.383 | 0.017 | 0.087 | -0.060 |
|  |  | CC53 | -0.075 | 0.111 | 0.229 | 0.112 | 0.466 | 0.590 | -0.051 | 0.077 | 0.041 | 0.292 | 0.055 | 0.034 | -0.034 | 0.700 | 0.486 | -0.016 | 0.095 | 0.004 |
|  |  | 4-1-31B | -0.050 | 0.136 | 0.263 | 0.108 | 0.392 | 0.277 | -0.069 | 0.049 | 0.064 | 0.311 | 0.191 | 0.030 | 0.025 | 0.667 | 0.513 | 0.032 | 0.112 | 0.008 |
|  |  | 3-1-36A2 | -0.030 | 0.117 | 0.143 | 0.088 | 0.459 | 0.458 | -0.002 | 0.052 | 0.048 | 0.225 | 0.076 | 0.204 | 0.081 | 0.824 | 0.465 | 0.001 | 0.079 | 0.012 |
|  |  | 3-1-36B3 | -0.206 | 0.119 | 0.150 | 0.084 | 0.794 | 0.809 | -0.177 | 0.107 | 0.034 | 0.496 | 0.061 | -0.007 | -0.017 | 0.981 | 0.790 | 0.158 | 0.070 | -0.121 |
|  |  | 1-1-2 | -0.099 | 0.012 | 0.124 | 0.077 | 0.567 | 0.406 | -0.082 | 0.190 | 0.058 | 0.250 | 0.058 | 0.043 | 0.093 | 0.921 | 0.455 | 0.121 | 0.090 | -0.064 |
|  |  | 4-1-13 | -0.161 | 0.168 | 0.091 | 0.129 | 0.695 | 0.215 | -0.144 | 0.060 | 0.078 | 0.511 | 0.261 | 0.052 | -0.005 | 0.596 | 0.248 | 0.365 | 0.088 | -0.119 |
|  |  | 3-1-27 | -0.010 | 0.139 | 0.201 | 0.135 | 0.462 | 0.446 | -0.029 | 0.074 | 0.091 | 0.244 | 0.146 | 0.167 | 0.079 | 0.745 | 0.463 | 0.161 | 0.113 | 0.035 |
| None | *F. animalis* | 1-A-7 | -1.157 | 0.036 | 0.140 | 0.039 | 0.575 | 1.000 | -1.162 | -0.713 | -0.271 | 0.307 | 0.534 | -0.516 | -0.912 | 0.874 | 0.472 | 0.577 | 0.026 | -1.100 |
|  |  | 1-A-36 | -1.181 | 0.169 | 0.198 | 0.049 | 0.840 | 1.000 | -1.115 | -0.752 | -0.200 | 0.656 | 0.602 | -0.375 | -0.997 | 1.000 | 0.897 | 0.674 | 0.403 | -1.142 |
|  |  | 2_6JVN3 | -0.904 | 0.388 | 0.391 | 0.188 | 0.757 | 1.000 | -0.845 | -0.474 | -0.182 | 0.456 | 0.512 | -0.159 | -0.988 | 0.734 | 0.487 | 0.541 | 0.555 | -0.808 |
|  |  | 2-A-37 | -0.997 | 0.184 | 0.149 | 0.036 | 0.836 | 1.000 | -1.001 | -0.601 | -0.239 | 0.664 | 0.734 | -0.452 | -1.189 | 0.768 | 0.584 | 0.480 | 0.424 | -0.934 |
|  |  | 7_33C1 | -1.119 | 0.082 | 0.185 | 0.117 | 0.726 | 1.000 | -1.084 | -0.808 | -0.233 | 0.638 | 0.881 | -0.477 | -1.227 | 0.939 | 0.641 | 0.412 | 0.265 | -1.079 |
|  |  | 3-1-37BFAA | -1.115 | 0.091 | 0.188 | 0.001 | 0.604 | 1.000 | -1.059 | -0.901 | -0.240 | 0.462 | 0.501 | -0.506 | -0.932 | 0.733 | 0.441 | 0.667 | 0.349 | -1.003 |
|  |  | 3-2-44B | -1.007 | 0.116 | 0.111 | 0.004 | 0.721 | 1.000 | -0.981 | -0.769 | -0.187 | 0.492 | 0.457 | -0.486 | -1.092 | 0.681 | 0.413 | 0.495 | 0.212 | -0.980 |
|  |  | 3-1-48A | -1.074 | 0.051 | 0.101 | 0.018 | 0.689 | 1.000 | -1.026 | -0.623 | -0.212 | 0.626 | 0.714 | -0.550 | -0.956 | 0.853 | 0.510 | 0.710 | 0.388 | -1.000 |
|  |  | 2-1-50A | -0.999 | 0.218 | 0.135 | -0.008 | 0.761 | 1.000 | -0.952 | -0.676 | -0.253 | 0.385 | 0.743 | -0.359 | -1.160 | 0.824 | 0.481 | 0.326 | 0.231 | -1.062 |
|  |  | 2-1-50B | -1.188 | 0.199 | 0.220 | 0.083 | 0.845 | 1.000 | -1.208 | -0.631 | -0.241 | 0.706 | 0.969 | -0.206 | -1.147 | 1.000 | 0.818 | 0.778 | 0.652 | -1.231 |
|  |  | 11-3-2 | -1.078 | 0.096 | 0.074 | -0.047 | 0.813 | 1.000 | -0.962 | -0.656 | -0.151 | 0.825 | 0.906 | -0.585 | -0.992 | 1.000 | 0.907 | 0.412 | -0.131 | -1.132 |
|  |  | 3-2-4 | -1.225 | 0.070 | 0.117 | 0.047 | 0.809 | 1.000 | -1.145 | -1.108 | -0.232 | 0.633 | 0.862 | -0.563 | -1.278 | 0.836 | 0.637 | 0.441 | 0.616 | -1.124 |
|  |  | 21-1A | -1.058 | 0.173 | 0.133 | -0.053 | 0.765 | 1.000 | -1.034 | -0.876 | -0.225 | 0.827 | 0.824 | -0.493 | -1.195 | 0.906 | 0.871 | 0.667 | 0.389 | -1.140 |
|  |  | 4-8 | -1.192 | 0.066 | 0.185 | 0.089 | 0.874 | 1.000 | -1.167 | -0.687 | -0.212 | 0.881 | 1.000 | -0.350 | -1.492 | 1.000 | 0.807 | 0.548 | 0.604 | -1.255 |
|  |  | 7-1 | -1.343 | 0.204 | 0.291 | 0.050 | 0.767 | 1.000 | -1.183 | -0.630 | -0.264 | 0.805 | 0.928 | -0.526 | -1.480 | 1.000 | 0.837 | 0.839 | 0.440 | -1.301 |
|  | *F. nucleatum* | 2_3FMU1 | -0.896 | 0.145 | 0.082 | -0.235 | 0.598 | 1.000 | -0.938 | -0.726 | -0.258 | 0.162 | 0.239 | -0.388 | -1.221 | 0.812 | 0.548 | 0.190 | -0.096 | -0.961 |
|  | *F. polymorphum* | 1-A-13 | -0.983 | 0.204 | 0.308 | -0.103 | 0.455 | 1.000 | -0.919 | -0.658 | -0.198 | 0.594 | 0.534 | -0.441 | -1.197 | 0.732 | 0.213 | 0.616 | -0.166 | -0.958 |
|  |  | 2-A-7 | -1.165 | 0.120 | 0.458 | -0.110 | 0.806 | 1.000 | -1.029 | -0.738 | -0.261 | 0.770 | 0.037 | -0.443 | -1.446 | 0.903 | 0.249 | 0.602 | 0.911 | -1.088 |
|  |  | 4-A-7 | -0.889 | 0.115 | 0.235 | 0.019 | 0.507 | 1.000 | -0.877 | -0.455 | -0.203 | 0.673 | 0.279 | -0.356 | -1.466 | 0.665 | 0.190 | 0.595 | 0.295 | -0.819 |
|  |  | 216A1 | -1.078 | 0.202 | 0.203 | 0.103 | 0.779 | 1.000 | -0.986 | -0.638 | -0.259 | 0.832 | 1.000 | -0.423 | -1.520 | 0.861 | 0.739 | 0.645 | 0.462 | -1.000 |
|  |  | 2-1-31 | -1.496 | 0.191 | 0.388 | 0.052 | 0.849 | 1.000 | -1.337 | -1.015 | -0.251 | 0.796 | 0.917 | -0.478 | -1.687 | 0.927 | 0.615 | 0.620 | 0.706 | -1.344 |
|  |  | 13-3C | -1.270 | 0.124 | 0.431 | 0.205 | 0.834 | 1.000 | -0.906 | -0.564 | -0.160 | 0.864 | 0.895 | -0.515 | -0.962 | 1.000 | 0.835 | 0.853 | 0.855 | -0.956 |
|  | *F. vincentii* | 2-A-13 | -0.987 | 0.204 | 0.216 | -0.050 | 0.701 | 1.000 | -0.873 | -0.825 | -0.194 | 0.641 | 0.684 | -0.205 | -1.127 | 0.833 | 0.758 | 0.509 | 0.409 | -0.912 |
|  |  | 3-A-6A | -1.071 | 0.097 | 0.091 | -0.021 | 0.723 | 1.000 | -1.009 | -0.960 | -0.241 | 0.760 | 0.664 | -0.277 | -0.968 | 0.828 | 0.805 | 0.495 | 0.521 | -0.995 |
|  |  | CC53 | -0.863 | 0.198 | 0.074 | -0.157 | 0.629 | 1.000 | -0.820 | -0.666 | -0.210 | 0.767 | 0.306 | -0.428 | -0.962 | 0.794 | 0.603 | 0.505 | -0.003 | -0.918 |
|  |  | 4-1-31B | -0.994 | 0.118 | 0.124 | 0.048 | 0.536 | 1.000 | -1.013 | -0.720 | -0.169 | 0.718 | 0.684 | -0.477 | -1.284 | 0.845 | 0.673 | 0.670 | 0.370 | -0.973 |
|  |  | 3-1-36A2 | -0.629 | 0.060 | 0.002 | -0.216 | 0.575 | 1.000 | -0.665 | -0.640 | -0.198 | 0.176 | -0.336 | -0.463 | -0.536 | 0.620 | 0.383 | 0.190 | -0.007 | -0.713 |
|  |  | 3-1-36B3 | -0.845 | 0.077 | -0.003 | -0.246 | 0.603 | 1.000 | -0.844 | -0.806 | -0.275 | 0.248 | 0.084 | -0.540 | -1.039 | 0.747 | 0.416 | 0.333 | -0.006 | -0.943 |
|  |  | 1-1-2 | -0.609 | 0.031 | 0.004 | -0.308 | 0.601 | 1.000 | -0.677 | -0.494 | -0.197 | 0.125 | -0.189 | -0.222 | -0.750 | 0.831 | 0.363 | 0.237 | -0.212 | -0.654 |
|  |  | 4-1-13 | -0.958 | 0.073 | 0.169 | -0.077 | 0.724 | 1.000 | -0.922 | -0.689 | -0.245 | 0.595 | 0.708 | -0.485 | -1.045 | 0.804 | 0.543 | 0.763 | 0.303 | -0.955 |
|  |  | 3-1-27 | -0.966 | 0.095 | 0.094 | -0.041 | 0.754 | 1.000 | -0.923 | -0.721 | -0.252 | 0.579 | 0.400 | -0.371 | -1.024 | 0.797 | 0.650 | 0.656 | 0.348 | -0.946 |
| Soy | *F. animalis* | 1-A-7 | -0.164 | 0.531 | 0.221 | 0.216 | 0.628 | 0.513 | 0.017 | 0.092 | 0.074 | 0.590 | 0.484 | 0.029 | 0.016 | 0.703 | 0.499 | 0.178 | 0.127 | 0.028 |
|  |  | 1-A-36 | -0.353 | 0.229 | 0.257 | 0.195 | 0.796 | 0.599 | -0.105 | 0.059 | 0.005 | 0.651 | 0.276 | 0.109 | -0.046 | 0.747 | 0.527 | 0.236 | 0.002 | -0.062 |
|  |  | 2_6JVN3 | -0.522 | 0.829 | 0.221 | 0.115 | 0.736 | 0.626 | -0.230 | 0.004 | 0.007 | 0.645 | 0.559 | 0.004 | -0.054 | 0.667 | 0.391 | 0.220 | 0.088 | -0.169 |
|  |  | 2-A-37 | -0.376 | 0.142 | 0.182 | 0.240 | 0.926 | 0.675 | -0.135 | 0.054 | 0.034 | 0.803 | 0.914 | 0.063 | 0.027 | 0.919 | 0.641 | 0.158 | 0.147 | -0.072 |
|  |  | 7_33C1 | -0.194 | 0.088 | 0.163 | 0.133 | 0.580 | 0.161 | -0.047 | 0.064 | 0.005 | 0.420 | 0.400 | 0.047 | 0.025 | 0.505 | 0.362 | 0.099 | 0.100 | -0.021 |
|  |  | 3-1-37BFAA | -0.368 | 0.136 | 0.202 | 0.109 | 0.670 | 0.295 | -0.151 | 0.239 | 0.012 | 0.541 | 0.414 | -0.015 | 0.018 | 0.583 | 0.336 | 0.000 | 0.026 | -0.115 |
|  |  | 3-2-44B | -0.213 | 0.241 | 0.165 | 0.160 | 0.726 | 0.300 | -0.065 | 0.106 | -0.015 | 0.587 | 0.505 | 0.043 | 0.032 | 0.622 | 0.360 | 0.290 | 0.115 | -0.039 |
|  |  | 3-1-48A | -0.248 | 0.117 | 0.150 | 0.128 | 0.673 | 0.241 | -0.091 | 0.076 | -0.004 | 0.624 | 0.409 | 0.019 | 0.033 | 0.594 | 0.362 | 0.072 | 0.049 | -0.078 |
|  |  | 2-1-50A | -0.240 | 0.222 | 0.139 | 0.058 | 0.598 | 0.549 | -0.140 | 0.164 | 0.027 | 0.539 | 0.597 | 0.094 | 0.056 | 0.618 | 0.378 | -0.012 | 0.106 | -0.128 |
|  |  | 2-1-50B | -0.287 | 0.247 | 0.269 | -0.032 | 0.907 | 0.681 | -0.138 | 0.081 | 0.046 | 0.534 | 0.574 | 0.027 | 0.034 | 0.975 | 0.588 | 0.271 | 0.085 | -0.082 |
|  |  | 11-3-2 | -0.389 | 0.054 | 0.156 | -0.076 | 0.903 | 0.431 | -0.214 | 0.071 | 0.023 | 0.517 | 0.332 | -0.030 | 0.027 | 0.951 | 0.691 | 0.116 | 0.017 | -0.203 |
|  |  | 3-2-4 | -0.217 | 0.249 | 0.055 | 0.099 | 0.717 | 0.306 | -0.089 | 0.121 | 0.004 | 0.652 | 0.499 | -0.024 | 0.003 | 0.688 | 0.279 | 0.147 | 0.042 | -0.093 |
|  |  | 21-1A | -0.345 | 0.090 | 0.129 | 0.121 | 0.777 | 0.555 | -0.172 | 0.186 | -0.005 | 0.663 | 0.347 | 0.036 | 0.003 | 0.793 | 0.499 | 0.150 | 0.071 | -0.128 |
|  |  | 4-8 | -0.385 | 0.077 | 0.184 | 0.212 | 0.936 | 0.644 | -0.172 | 0.091 | 0.005 | 0.985 | 1.000 | 0.032 | -0.001 | 0.992 | 0.990 | 0.224 | 0.124 | -0.124 |
|  |  | 7-1 | -0.311 | 0.055 | 0.465 | -0.041 | 0.835 | 0.540 | -0.014 | 0.074 | -0.003 | 0.819 | 0.801 | 0.182 | 0.030 | 0.918 | 0.745 | 0.270 | 0.045 | 0.049 |
|  | *F. nucleatum* | 2_3FMU1 | -0.211 | 0.258 | 0.055 | 0.010 | 0.285 | 0.479 | -0.133 | -0.055 | 0.013 | 0.679 | 0.115 | 0.003 | 0.017 | 0.811 | 0.587 | -0.241 | 0.006 | -0.126 |
|  | *F. polymorphum* | 1-A-13 | -0.236 | 0.089 | 0.145 | -0.024 | 0.143 | 0.494 | -0.088 | 0.138 | 0.007 | 0.271 | 0.019 | 0.051 | 0.044 | 0.335 | 0.048 | -0.007 | -0.008 | -0.070 |
|  |  | 2-A-7 | -0.261 | 0.142 | 0.128 | 0.029 | 0.781 | 0.262 | -0.128 | 0.013 | -0.007 | 0.621 | 0.024 | -0.001 | -0.004 | 0.329 | 0.024 | 0.214 | 0.147 | -0.097 |
|  |  | 4-A-7 | -0.080 | 0.115 | 0.069 | 0.088 | 0.140 | 0.031 | -0.043 | 0.008 | 0.012 | 0.141 | 0.100 | 0.050 | 0.035 | 0.200 | 0.057 | 0.020 | 0.015 | -0.023 |
|  |  | 216A1 | -0.300 | 0.081 | 0.216 | 0.169 | 0.672 | 0.400 | -0.086 | 0.033 | 0.040 | 0.622 | 0.760 | 0.036 | -0.010 | 0.717 | 0.571 | 0.274 | 0.039 | -0.072 |
|  |  | 2-1-31 | -0.680 | 0.290 | 0.570 | -0.068 | 0.553 | 1.000 | -0.201 | 0.048 | -0.001 | 0.979 | 0.462 | -0.005 | -0.069 | 0.635 | 0.119 | 0.447 | 0.091 | -0.112 |
|  |  | 13-3C | -0.267 | 0.044 | 0.043 | -0.001 | 0.041 | 0.013 | -0.138 | 0.005 | 0.009 | 0.069 | 0.019 | -0.036 | 0.015 | 0.301 | 0.002 | -0.162 | 0.046 | -0.135 |
|  | *F. vincentii* | 2-A-13 | -0.215 | 0.315 | 0.227 | 0.093 | 0.675 | 0.571 | -0.047 | 0.070 | 0.026 | 0.588 | 0.410 | 0.055 | 0.012 | 0.702 | 0.520 | -0.060 | 0.079 | -0.036 |
|  |  | 3-A-6A | -0.224 | 0.201 | 0.038 | 0.084 | 0.821 | 0.628 | -0.109 | 0.010 | 0.023 | 0.623 | 0.238 | 0.017 | -0.001 | 0.772 | 0.314 | -0.292 | 0.022 | -0.115 |
|  |  | CC53 | -0.177 | 0.105 | 0.179 | 0.110 | 0.494 | 0.384 | -0.045 | 0.061 | 0.036 | 0.435 | 0.039 | 0.001 | 0.024 | 0.638 | 0.455 | 0.223 | 0.022 | -0.035 |
|  |  | 4-1-31B | -0.260 | 0.032 | 0.109 | 0.094 | 0.451 | 0.283 | -0.072 | -0.018 | 0.003 | 0.420 | 0.234 | -0.059 | 0.014 | 0.658 | 0.331 | 0.144 | 0.036 | -0.063 |
|  |  | 3-1-36A2 | -0.147 | 0.104 | 0.113 | 0.102 | 0.725 | 0.426 | -0.041 | 0.154 | 0.031 | 0.317 | 0.081 | 0.042 | 0.075 | 0.860 | 0.445 | -0.019 | 0.081 | -0.042 |
|  |  | 3-1-36B3 | -0.331 | 0.148 | 0.100 | 0.064 | 0.906 | 0.743 | -0.165 | 0.058 | 0.000 | 0.686 | 0.133 | -0.018 | -0.007 | 0.984 | 0.787 | 0.101 | 0.070 | -0.136 |
|  |  | 1-1-2 | -0.202 | 0.022 | 0.067 | 0.082 | 0.654 | 0.535 | -0.099 | 0.039 | 0.007 | 0.310 | 0.081 | 0.027 | 0.013 | 0.755 | 0.406 | 0.020 | 0.106 | -0.073 |
|  |  | 4-1-13 | -0.199 | 0.122 | 0.239 | 0.147 | 0.563 | 0.277 | -0.062 | 0.070 | 0.031 | 0.501 | 0.503 | 0.047 | 0.058 | 0.558 | 0.504 | 0.096 | 0.070 | -0.016 |
|  |  | 3-1-27 | -0.215 | 0.177 | 0.066 | 0.105 | 0.515 | 0.401 | -0.113 | -0.037 | 0.087 | 0.379 | 0.138 | 0.031 | 0.021 | 0.580 | 0.337 | 0.341 | 0.067 | -0.103 |

**Table S5:** Genomes and accession numbers used for cladal analysis.

| **Strain** | **Bioproject** |
| --- | --- |
| 1-A-36 | PRJNA1140621 |
| 3-1-37BFAA | PRJNA1140621 |
| 7_33C1 | PRJNA1140621 |
| 4-8 | PRJNA32481 |
| 2-1-50B | PRJNA32501 |
| 21-1A | PRJNA32475 |
| 2-A-37 | PRJNA1140621 |
| 2_6JVN3 | PRJNA1140621 |
| 7-1 | PRJNA32483 |
| 11-3-2 | PRJNA39559 |

**Table S6:** Significant differences in amino acid utilization averaged across strains within *Fusobacterium* spp. amino acid metabolism clusters. *Fusobacterium* spp. clusters were created by k-means clustering of amino acid utilization profiles. Significance in mean percent utilization across all 18 amino acids detected between each cluster were evaluated using two-way ANOVA followed by Tukey’s Honest Significant Difference post-hoc tests (Tukey’s HSD). Adjusted p-values are indicated with asterisks: **** = p-value < 0.0001, *** = p-value < 0.001, ** = p-value < 0.01, * = p-value < 0.05, ns = not significant.

| Amino acid | Fusobacterium spp. clusters compared by Tukey's multiple comparisons test | Adjusted p-value |
| --- | --- | --- |
| **Alanine** | Cluster 1 vs. Cluster 2 | * |
|  | Cluster 1 vs. Cluster 3 | ns |
|  | Cluster 1 vs. Cluster 4 | ** |
|  | Cluster 1 vs. Cluster 5 | ** |
|  | Cluster 1 vs. Cluster 6 | ns |
|  | Cluster 2 vs. Cluster 3 | **** |
|  | Cluster 2 vs. Cluster 4 | **** |
|  | Cluster 2 vs. Cluster 5 | **** |
|  | Cluster 2 vs. Cluster 6 | *** |
|  | Cluster 3 vs. Cluster 4 | ns |
|  | Cluster 3 vs. Cluster 5 | ns |
|  | Cluster 3 vs. Cluster 6 | ns |
|  | Cluster 4 vs. Cluster 5 | ns |
|  | Cluster 4 vs. Cluster 6 | * |
|  | Cluster 5 vs. Cluster 6 | ns |
| **Arginine** | Cluster 1 vs. Cluster 2 | **** |
|  | Cluster 1 vs. Cluster 3 | ns |
|  | Cluster 1 vs. Cluster 4 | ns |
|  | Cluster 1 vs. Cluster 5 | ns |
|  | Cluster 1 vs. Cluster 6 | ns |
|  | Cluster 2 vs. Cluster 3 | **** |
|  | Cluster 2 vs. Cluster 4 | **** |
|  | Cluster 2 vs. Cluster 5 | **** |
|  | Cluster 2 vs. Cluster 6 | **** |
|  | Cluster 3 vs. Cluster 4 | ns |
|  | Cluster 3 vs. Cluster 5 | ns |
|  | Cluster 3 vs. Cluster 6 | ns |
|  | Cluster 4 vs. Cluster 5 | ns |
|  | Cluster 4 vs. Cluster 6 | ns |
|  | Cluster 5 vs. Cluster 6 | ns |
| **Asparagine** | Cluster 1 vs. Cluster 2 | * |
|  | Cluster 1 vs. Cluster 3 | ns |
|  | Cluster 1 vs. Cluster 4 | * |
|  | Cluster 1 vs. Cluster 5 | ** |
|  | Cluster 1 vs. Cluster 6 | ns |
|  | Cluster 2 vs. Cluster 3 | **** |
|  | Cluster 2 vs. Cluster 4 | **** |
|  | Cluster 2 vs. Cluster 5 | **** |
|  | Cluster 2 vs. Cluster 6 | **** |
|  | Cluster 3 vs. Cluster 4 | ns |
|  | Cluster 3 vs. Cluster 5 | ns |
|  | Cluster 3 vs. Cluster 6 | ns |
|  | Cluster 4 vs. Cluster 5 | ns |
|  | Cluster 4 vs. Cluster 6 | ns |
|  | Cluster 5 vs. Cluster 6 | ns |
| **Aspartate** | Cluster 1 vs. Cluster 2 | * |
|  | Cluster 1 vs. Cluster 3 | ns |
|  | Cluster 1 vs. Cluster 4 | ** |
|  | Cluster 1 vs. Cluster 5 | ** |
|  | Cluster 1 vs. Cluster 6 | * |
|  | Cluster 2 vs. Cluster 3 | ns |
|  | Cluster 2 vs. Cluster 4 | ns |
|  | Cluster 2 vs. Cluster 5 | ns |
|  | Cluster 2 vs. Cluster 6 | ns |
|  | Cluster 3 vs. Cluster 4 | * |
|  | Cluster 3 vs. Cluster 5 | ns |
|  | Cluster 3 vs. Cluster 6 | ns |
|  | Cluster 4 vs. Cluster 5 | ns |
|  | Cluster 4 vs. Cluster 6 | ns |
|  | Cluster 5 vs. Cluster 6 | ns |
| **Glutamate** | Cluster 1 vs. Cluster 2 | ns |
|  | Cluster 1 vs. Cluster 3 | *** |
|  | Cluster 1 vs. Cluster 4 | **** |
|  | Cluster 1 vs. Cluster 5 | **** |
|  | Cluster 1 vs. Cluster 6 | ns |
|  | Cluster 2 vs. Cluster 3 | ns |
|  | Cluster 2 vs. Cluster 4 | **** |
|  | Cluster 2 vs. Cluster 5 | **** |
|  | Cluster 2 vs. Cluster 6 | ns |
|  | Cluster 3 vs. Cluster 4 | **** |
|  | Cluster 3 vs. Cluster 5 | **** |
|  | Cluster 3 vs. Cluster 6 | * |
|  | Cluster 4 vs. Cluster 5 | **** |
|  | Cluster 4 vs. Cluster 6 | **** |
|  | Cluster 5 vs. Cluster 6 | **** |
| **Glutamine** | Cluster 1 vs. Cluster 2 | **** |
|  | Cluster 1 vs. Cluster 3 | **** |
|  | Cluster 1 vs. Cluster 4 | **** |
|  | Cluster 1 vs. Cluster 5 | **** |
|  | Cluster 1 vs. Cluster 6 | ns |
|  | Cluster 2 vs. Cluster 3 | **** |
|  | Cluster 2 vs. Cluster 4 | **** |
|  | Cluster 2 vs. Cluster 5 | **** |
|  | Cluster 2 vs. Cluster 6 | **** |
|  | Cluster 3 vs. Cluster 4 | ** |
|  | Cluster 3 vs. Cluster 5 | * |
|  | Cluster 3 vs. Cluster 6 | **** |
|  | Cluster 4 vs. Cluster 5 | **** |
|  | Cluster 4 vs. Cluster 6 | **** |
|  | Cluster 5 vs. Cluster 6 | **** |
| **Glycine** | Cluster 1 vs. Cluster 2 | ns |
|  | Cluster 1 vs. Cluster 3 | ns |
|  | Cluster 1 vs. Cluster 4 | ns |
|  | Cluster 1 vs. Cluster 5 | ns |
|  | Cluster 1 vs. Cluster 6 | ns |
|  | Cluster 2 vs. Cluster 3 | * |
|  | Cluster 2 vs. Cluster 4 | * |
|  | Cluster 2 vs. Cluster 5 | * |
|  | Cluster 2 vs. Cluster 6 | ns |
|  | Cluster 3 vs. Cluster 4 | ns |
|  | Cluster 3 vs. Cluster 5 | ns |
|  | Cluster 3 vs. Cluster 6 | ns |
|  | Cluster 4 vs. Cluster 5 | ns |
|  | Cluster 4 vs. Cluster 6 | ns |
|  | Cluster 5 vs. Cluster 6 | ns |
| **Isoleucine** | Cluster 1 vs. Cluster 2 | ns |
|  | Cluster 1 vs. Cluster 3 | ns |
|  | Cluster 1 vs. Cluster 4 | ns |
|  | Cluster 1 vs. Cluster 5 | ns |
|  | Cluster 1 vs. Cluster 6 | ns |
|  | Cluster 2 vs. Cluster 3 | ns |
|  | Cluster 2 vs. Cluster 4 | ns |
|  | Cluster 2 vs. Cluster 5 | ns |
|  | Cluster 2 vs. Cluster 6 | ns |
|  | Cluster 3 vs. Cluster 4 | ns |
|  | Cluster 3 vs. Cluster 5 | ns |
|  | Cluster 3 vs. Cluster 6 | ns |
|  | Cluster 4 vs. Cluster 5 | ns |
|  | Cluster 4 vs. Cluster 6 | ns |
|  | Cluster 5 vs. Cluster 6 | ns |
| **Leucine** | Cluster 1 vs. Cluster 2 | ns |
|  | Cluster 1 vs. Cluster 3 | ns |
|  | Cluster 1 vs. Cluster 4 | ns |
|  | Cluster 1 vs. Cluster 5 | ns |
|  | Cluster 1 vs. Cluster 6 | ns |
|  | Cluster 2 vs. Cluster 3 | ns |
|  | Cluster 2 vs. Cluster 4 | ns |
|  | Cluster 2 vs. Cluster 5 | ns |
|  | Cluster 2 vs. Cluster 6 | ns |
|  | Cluster 3 vs. Cluster 4 | ns |
|  | Cluster 3 vs. Cluster 5 | ns |
|  | Cluster 3 vs. Cluster 6 | ns |
|  | Cluster 4 vs. Cluster 5 | ns |
|  | Cluster 4 vs. Cluster 6 | ns |
|  | Cluster 5 vs. Cluster 6 | ns |
| **Lysine** | Cluster 1 vs. Cluster 2 | ns |
|  | Cluster 1 vs. Cluster 3 | **** |
|  | Cluster 1 vs. Cluster 4 | **** |
|  | Cluster 1 vs. Cluster 5 | **** |
|  | Cluster 1 vs. Cluster 6 | **** |
|  | Cluster 2 vs. Cluster 3 | **** |
|  | Cluster 2 vs. Cluster 4 | **** |
|  | Cluster 2 vs. Cluster 5 | **** |
|  | Cluster 2 vs. Cluster 6 | **** |
|  | Cluster 3 vs. Cluster 4 | **** |
|  | Cluster 3 vs. Cluster 5 | **** |
|  | Cluster 3 vs. Cluster 6 | ns |
|  | Cluster 4 vs. Cluster 5 | ** |
|  | Cluster 4 vs. Cluster 6 | **** |
|  | Cluster 5 vs. Cluster 6 | **** |
| **Methionine** | Cluster 1 vs. Cluster 2 | **** |
|  | Cluster 1 vs. Cluster 3 | **** |
|  | Cluster 1 vs. Cluster 4 | **** |
|  | Cluster 1 vs. Cluster 5 | **** |
|  | Cluster 1 vs. Cluster 6 | **** |
|  | Cluster 2 vs. Cluster 3 | ns |
|  | Cluster 2 vs. Cluster 4 | **** |
|  | Cluster 2 vs. Cluster 5 | **** |
|  | Cluster 2 vs. Cluster 6 | ns |
|  | Cluster 3 vs. Cluster 4 | **** |
|  | Cluster 3 vs. Cluster 5 | **** |
|  | Cluster 3 vs. Cluster 6 | ns |
|  | Cluster 4 vs. Cluster 5 | ns |
|  | Cluster 4 vs. Cluster 6 | *** |
|  | Cluster 5 vs. Cluster 6 | ** |
| **Phenylalanine** | Cluster 1 vs. Cluster 2 | ns |
|  | Cluster 1 vs. Cluster 3 | ns |
|  | Cluster 1 vs. Cluster 4 | ns |
|  | Cluster 1 vs. Cluster 5 | ns |
|  | Cluster 1 vs. Cluster 6 | ns |
|  | Cluster 2 vs. Cluster 3 | ns |
|  | Cluster 2 vs. Cluster 4 | ns |
|  | Cluster 2 vs. Cluster 5 | ns |
|  | Cluster 2 vs. Cluster 6 | ns |
|  | Cluster 3 vs. Cluster 4 | ns |
|  | Cluster 3 vs. Cluster 5 | ns |
|  | Cluster 3 vs. Cluster 6 | ns |
|  | Cluster 4 vs. Cluster 5 | ns |
|  | Cluster 4 vs. Cluster 6 | ns |
|  | Cluster 5 vs. Cluster 6 | ns |
| **Proline** | Cluster 1 vs. Cluster 2 | ns |
|  | Cluster 1 vs. Cluster 3 | ns |
|  | Cluster 1 vs. Cluster 4 | ns |
|  | Cluster 1 vs. Cluster 5 | ns |
|  | Cluster 1 vs. Cluster 6 | ns |
|  | Cluster 2 vs. Cluster 3 | ns |
|  | Cluster 2 vs. Cluster 4 | ns |
|  | Cluster 2 vs. Cluster 5 | ns |
|  | Cluster 2 vs. Cluster 6 | ns |
|  | Cluster 3 vs. Cluster 4 | ns |
|  | Cluster 3 vs. Cluster 5 | ns |
|  | Cluster 3 vs. Cluster 6 | ns |
|  | Cluster 4 vs. Cluster 5 | ns |
|  | Cluster 4 vs. Cluster 6 | ns |
|  | Cluster 5 vs. Cluster 6 | ns |
| **Serine** | Cluster 1 vs. Cluster 2 | ** |
|  | Cluster 1 vs. Cluster 3 | **** |
|  | Cluster 1 vs. Cluster 4 | **** |
|  | Cluster 1 vs. Cluster 5 | *** |
|  | Cluster 1 vs. Cluster 6 | ns |
|  | Cluster 2 vs. Cluster 3 | ns |
|  | Cluster 2 vs. Cluster 4 | **** |
|  | Cluster 2 vs. Cluster 5 | ns |
|  | Cluster 2 vs. Cluster 6 | *** |
|  | Cluster 3 vs. Cluster 4 | **** |
|  | Cluster 3 vs. Cluster 5 | ns |
|  | Cluster 3 vs. Cluster 6 | **** |
|  | Cluster 4 vs. Cluster 5 | **** |
|  | Cluster 4 vs. Cluster 6 | **** |
|  | Cluster 5 vs. Cluster 6 | **** |
| **Threonine** | Cluster 1 vs. Cluster 2 | **** |
|  | Cluster 1 vs. Cluster 3 | **** |
|  | Cluster 1 vs. Cluster 4 | **** |
|  | Cluster 1 vs. Cluster 5 | **** |
|  | Cluster 1 vs. Cluster 6 | ns |
|  | Cluster 2 vs. Cluster 3 | ns |
|  | Cluster 2 vs. Cluster 4 | * |
|  | Cluster 2 vs. Cluster 5 | ns |
|  | Cluster 2 vs. Cluster 6 | **** |
|  | Cluster 3 vs. Cluster 4 | **** |
|  | Cluster 3 vs. Cluster 5 | ns |
|  | Cluster 3 vs. Cluster 6 | **** |
|  | Cluster 4 vs. Cluster 5 | **** |
|  | Cluster 4 vs. Cluster 6 | **** |
|  | Cluster 5 vs. Cluster 6 | **** |
| **Tryptophan** | Cluster 1 vs. Cluster 2 | ns |
|  | Cluster 1 vs. Cluster 3 | ** |
|  | Cluster 1 vs. Cluster 4 | **** |
|  | Cluster 1 vs. Cluster 5 | **** |
|  | Cluster 1 vs. Cluster 6 | *** |
|  | Cluster 2 vs. Cluster 3 | * |
|  | Cluster 2 vs. Cluster 4 | **** |
|  | Cluster 2 vs. Cluster 5 | **** |
|  | Cluster 2 vs. Cluster 6 | ** |
|  | Cluster 3 vs. Cluster 4 | * |
|  | Cluster 3 vs. Cluster 5 | ** |
|  | Cluster 3 vs. Cluster 6 | ns |
|  | Cluster 4 vs. Cluster 5 | ns |
|  | Cluster 4 vs. Cluster 6 | ns |
|  | Cluster 5 vs. Cluster 6 | ns |
| **Tyrosine** | Cluster 1 vs. Cluster 2 | ns |
|  | Cluster 1 vs. Cluster 3 | ns |
|  | Cluster 1 vs. Cluster 4 | ns |
|  | Cluster 1 vs. Cluster 5 | ns |
|  | Cluster 1 vs. Cluster 6 | ns |
|  | Cluster 2 vs. Cluster 3 | ns |
|  | Cluster 2 vs. Cluster 4 | ns |
|  | Cluster 2 vs. Cluster 5 | ns |
|  | Cluster 2 vs. Cluster 6 | ns |
|  | Cluster 3 vs. Cluster 4 | ns |
|  | Cluster 3 vs. Cluster 5 | ns |
|  | Cluster 3 vs. Cluster 6 | ns |
|  | Cluster 4 vs. Cluster 5 | ns |
|  | Cluster 4 vs. Cluster 6 | ns |
|  | Cluster 5 vs. Cluster 6 | ns |
| **Valine** | Cluster 1 vs. Cluster 2 | ns |
|  | Cluster 1 vs. Cluster 3 | ns |
|  | Cluster 1 vs. Cluster 4 | ns |
|  | Cluster 1 vs. Cluster 5 | ns |
|  | Cluster 1 vs. Cluster 6 | ns |
|  | Cluster 2 vs. Cluster 3 | ns |
|  | Cluster 2 vs. Cluster 4 | ns |
|  | Cluster 2 vs. Cluster 5 | ns |
|  | Cluster 2 vs. Cluster 6 | ns |
|  | Cluster 3 vs. Cluster 4 | ns |
|  | Cluster 3 vs. Cluster 5 | ns |
|  | Cluster 3 vs. Cluster 6 | ns |
|  | Cluster 4 vs. Cluster 5 | ns |
|  | Cluster 4 vs. Cluster 6 | ns |
|  | Cluster 5 vs. Cluster 6 | ns |
